# Structural and functional covariance architecture of major depressive disorder: A meta-analytic structural equation modeling approach to primary neuroimaging analysis

**DOI:** 10.1016/j.bosn.2025.04.008

**Published:** 2025-05-01

**Authors:** Jodie P. Gray, Larry R. Price, Crystal Franklin, Cassandra D. Leonardo, Florence L. Chiang, Ki Sueng Choi, John Blangero, David C. Glahn, Helen S. Mayberg, Peter T. Fox

**Affiliations:** a Research Imaging Institute, University of Texas Health San Antonio, Radiology, UTHSCSA, San Antonio, TX, United States; b Department of Mathematics and Education, Texas State University, San Marcos, TX, United States; c Departments of Neurology and Neurosurgery, Icahn School of Medicine at Mount Sinai, New York, NY, United States; d Department of Human Genetics, University of Texas Rio Grande Valley School of Medicine, Brownsville, TX, United States; e South Texas Diabetes and Obesity Institute, University of Texas Rio Grande Valley School of Medicine, Brownsville, TX, United States; f Department of Psychiatry, Boston Children’s Hospital and Harvard Medical School, Boston, MA, United States; g Olin Neuropsychiatric Research Center, Institute of Living, Hartford Hospital, Hartford, CT, United States; h Department of Psychiatry and Behavioral Sciences, Emory University School of Medicine, Atlanta, Georgia; i Research Service, South Texas Veterans Administration Medical Center, San Antonio, TX, United States

**Keywords:** Major depressive disorder (MDD), Neuroimaging, Meta-analysis, Structural equation modeling, Network modeling, Node-and-edge networks

## Abstract

Neuroimaging studies of major depressive disorder (MDD) report widespread disease-attributed abnormalities of brain structure and function. However, reports from mass univariate-driven studies are inconsistent. The objective of this study was to determine if a neuroimaging-based biomarker of MDD, which can reliably distinguish patients from healthy controls, can be generated using multivariate measures. Multivariate modeling of MDD was achieved through generation of a meta-analytic node-and-edge network model of MDD in which disease impacted brain regions (nodes) and their covariances (edges) were quantified with structural equation modeling (SEM). SEM assessment and voxel-based morphometry (VBM) analysis in primary datasets served to test our hypothesis that multivariate analyses of MDD provide improved signal over mass univariate methods. Brain areas reliably impacted by MDD (nodes) and their covariances (edges) were informed by previously published coordinate-based meta-analysis activation/anatomical likelihood estimation (CBMA-ALE) by our group. Meta-analytic model was then fit in primary structural (T1) magnetic resonance imaging (MRI) data and resting-state functional MRI (rs-fMRI) data. Primary datasets were derived from two previously recruited cohorts. Outcome measures (testing for differences between MDD and controls) from standardized SEM included: a) model goodness of fit assessment, and b) individual edge strength. SEM measures were assessed in heterogeneous MDD patient groups, and subsequently re-tested in 7 clinical subgroups of MDD patients. Meta-analytically generated MDD network model yielded 9 nodes with 6 edges among the regions. Model goodness of fit in meta-analytic datasets were good to exceptional. Model goodness of fit in regionally sampled gray matter density in primary T1 data was exceptional in clinical subgroups of MDD, poor in clinically heterogeneous subgroups of MDD, and poor in healthy control subjects. VBM analysis of the same T1 datasets yielded sparse results. Model goodness did not distinguish MDD from controls in regionally sampled primary rs-fMRI. These findings support our hypothesis of improved multivariate signal in MDD compared to findings derived from mass univariate analyses, however this effect was only detectable in T1 data (groupwise). Improved SEM goodness of fit in clinical subgroups of MDD patients supports our hypothesis of detectable neuroimaging effects of clinical heterogeneity in MDD.

## 1. Introduction

Major depressive disorder (MDD) impacts over 300 million people annually, causing significant impairment in sufferers’ quality of life and functioning (World Health Organization, 2020). Neuroimaging offers powerful tools to non-invasively investigate disruptions of brain structure and function associated with neuropsychiatric disorders (Masdeu, 2011; Kupfer et al., 2012). The wide availability of data from published neuroimaging studies offers the opportunity to probe the literature for reliable pooled signal across primary clinical studies through big-data driven meta-analysis (Fox et al., 2014; Samartsidis et al., 2017). Previous studies by our group include coordinate-based meta-analysis (CMBA) (Fox et al., 2014) of both voxel-based morphometry (structural) and voxel-based physiology (task-independent functional) studies of MDD (Gray et al., 2020, 2022). Findings from these works demonstrate spatially convergent disease-specific abnormalities in MDD, of which convergence was improved by delineating patient groups by symptom category. The present study aims to expand on previous work by identifying a quantifiable, predictive, network-based biomarker of MDD among brain regions determined to be reliably impacted by the disease.

Disease-specific network modeling in MDD has early origins in work by Helen Mayberg (Mayberg,1997). The limbic-cortical model proposed by Mayberg was among the earliest to propose a heuristic, networked model of brain aberrations for use in future investigation of neurobiological mechanisms underlying depression. Dysregulated networks as drivers of MDD symptoms underpinned the basis for network-based treatment approaches such as deep brain stimulation (DBS) and transcranial magnetic stimulation (TMS) (Mayberg et al., 2005; Johansen-Berg et al., 2008; Lozano et al., 2008; Naurshima et al., 2010; Fox et al., 2012; Hadas et al., 2019; Singh et al., 2019). Since the work pioneered by Mayberg, increasingly powerful neuroimaging techniques have allowed for deeper investigations into the pathogenesis of disease. However, the search for disease-specific effects of depression overshot the mark in task-activation based neuroimaging studies, in which there can be both bias in the task (Fields et al., 2019) and a complete failure of convergence in task-activation meta-analysis (Müller et al., 2017).

Task-independent voxel-based physiology (VBP) (Drevets et al., 1997) and voxel-based morphometry (VBM) (Smith et al., 2004) offer whole-brain, mass univariate investigation of disease effects while mitigating the methodological challenges of task-activation studies. The task-independent framework allowed for the influential VBM study by David Glahn and colleagues in 2008 (Glahn et al., 2008) which reliably elucidated neuroanatomical disruptions in schizophrenia via meta-analysis. Following the ten-year span between the seminal work of Mayberg and the work of Glahn and colleagues, the literature rapidly expanded with a wealth of whole-brain, connectomic-based investigations of neuropsychiatric disorders. Work by Seeley et al. presented the research community with the network degeneration hypothesis (NDH), through which disease effects were proposed to propagate through brain networks via several posited biological mechanisms (Seeley et al., 2009). Following the theory posed by the NDH, several studies set out to test for connectomically-organized brain abnormalities shared across related neuropsychiatric disorders (Crossley et al., 2014; Vanasse et al., 2018; Goodkind et al., 2015; Saxena and Caroni, 2011; Zhou et al., 2012; Cioli et al., 2014; Ahmed et al., 2016; Brettschneider et al., 2015; Iturria-Medina et al., 2014; Warren et al., 2013; Cohen-Cory et al., 2010; Appel, 1981). The collective findings from these studies indicate that, transdiagnostically, many disorders share commonly impacted “hub” brain regions, lending support for the metabolic stress mechanism of the NDH. Shared effects across disorders also provide motivation for the next tier of analysis in which disease-specific effects may only be discernable from multivariate analysis of the commonly shared brain aberrations (Winter et al., 2022; Cauda et al., 2018a, 2018b, 2019; Manuello et al., 2018; Gray et al., 2022). The next immediate challenge for network modeling is capturing the signature of individual disorders, with increased difficulty in MDD due to the high level of heterogeneity in symptomology.

Network-model generation through meta-analysis offers a powerful tool to allow heuristic disease models to arise from a data-driven approach. Previous work by our group on MDD (Gray et al., (2020); Gray et al., (2022) and others (Barron et al., 2013; Kotkowski et al., 2018; Chiang et al., 2019) demonstrate the utility of using coordinate-based meta-analysis (CBMA) for model building and preserving primary neuroimaging datasets for model validation. Structural equation modeling (SEM) further extends the utility of CBMA by modeling impacted brain areas as “nodes” and their covariances as quantifiable connected “edges” (McLntosh et al., 1994). Due to the heterogeneity of MDD, a central hypothesis underlying the present study is that our data-driven modeling approach may allow clusters, observable through quantified SEM model fits, to arise directly from the neuroimaging analysis. We propose that heuristic clustering from imaging data may inform symptom clustering in subsequent analyses, with an ultimate goal towards establishing future correlates with clinical interventions.

The aim of the present study is to generate a meta-analytically derived SEM (“meta-SEM”) network model of depression and apply the model to structural and functional magnetic resonance imaging (MRI) primary data from previously recruited MDD patient cohorts, testing model goodness of fit with conventional SEM metrics. Based on indications of clinical heterogeneity impacts in neuroimaging convergence reported in by our group’s previous work, a central aim is testing the proposed depression model in subgroups of patients to the furthest extent possible given the reported clinical characteristics of the sampled population.

Our overarching hypothesis is that the meta-analytic-to-test pipeline for meta-SEM network-model generation will yield a suitable network model of MDD. Our first hypothesis (1a) is that the meta-SEM model will fit primary structural MRI data and (1b) primary functional (fMRI) data. Our secondary hypothesis is that the meta-SEM model will demonstrate good model fit in MDD patient primary data and will fit poorly in healthy control subject primary data in both (2a) structural MRI and (2b) fMRI data. Model fit will be assessed using standardized SEM measures of goodness-of-fit and quantized edge weights. Third, we hypothesize that differences in meta-SEM model fit will distinguish MDD patients from healthy control subjects both at the groupwise level (structural and fMRI) and at the per-subject level (testable in fMRI only). Fourth, per findings from our previous work, we hypothesize that clinical heterogeneity effects of depression will be detectable when network models are applied to clinical subgroups of depression patients, as demonstrated by overall model goodness-of-fit and differences in edge weights. Finally, we hypothesize that network-based (multivariate) effects of MDD will be stronger than effects derived from mass-univariate (voxel-based morphometry, VBM) analyses of the same primary structural MRI datasets.

## 2. Methods

### 2.1. Coordinate-based Meta-analysis

The meta-analytic dataset used in the present study was derived from previously published coordinate-based meta-analysis (CBMA) of MDD conducted by our group (Gray et al., 2020). The dataset includes coordinate-based findings (foci) from 92 voxel-wise, whole-brain neuroimaging studies in depression spanning voxel-based morphometry (VBM), amplitude of low frequency fluctuations (ALFF), regional homogeneity (ReHo), arterial spin labeling (ASL), positron emission tomography (PET) and single photon emission tomography (SPECT) investigations. Studies included in this dataset represent disease-control contrast findings from 2928 MDD patients and 2739 healthy control subjects. Foci from all studies– regardless of imaging modality or sign of observed effect– were combined into a unified, multi-modality group for analysis. See supplemental materials for included studies.

### 2.2. Node discovery

Nodes were identified using a modified version of the activation likelihood estimation (ALE) algorithm (Eickhoff et al., 2009, 2012; Turkeltaub et al., 2002; Laird et al., 2005), facilitated through use of GingerALE software (GingerALE edition 3.0, 2019). The unthresholded ALE union map of all coordinates reported from the previously described 92 studies was used in the modified ALE procedure. Peak regions from the unthresholded ALE map were selected using a method modified from Cauda et al. (Cauda et al., 2018a, 2018b, 2019; Manuello et al., 2018; Gray et al., 2022), which creates a histogram of all of the ALE values in the unthresholded map and allows the top percentile of peak ALE values to be retained as nodes. For the present study, the routine from Cauda et al. was modified to allow for the top 0.5 % of peaks to be selected as node regions.

### 2.3. Edge discovery

Edge discovery was achieved through previously published functional meta-analytic connectivity modeling (fMACM) methods (Chiang et al., 2019; Robinson et al., 2010; Robinson et al., 2012; Reid et al.; 2017). For each node identified in the previous step, a 10 mm diameter spherical region of interest, centered at the peak X,Y,Z Talairach coordinate for the node, was created using Mango (Mango version 4.1) software. Each ROI image was then used as a seed region for sampling within the BrainMap database (Fox and Lancaster, 2002), searching only in Normal Subjects All-Activations data. Sampling each seed in the database yielded each seed its own ALE map, which was subsequently transformed to Z score values. For each seed map, all other regions (nodes) identified in the previous step were sampled using 10 mm diameter ROIs to assess potential connectivity between seed and other nodes. A seed-to-region connection (edge) was retained if the average Z score within a sampled region ROI was at least 3.0. For instances of bidirectional connectivity, the seed-to-region score that had the greater average Z score was used as the edge’s directionality for subsequent SEM.

### 2.4. Primary data cohorts

Primary patient data was sourced from three previously acquired cohorts: 1) The Genetics of Brain Structure and Function Study (GOBS) (Winkler et al., 2010) and 2) two cohorts from studies conducted by Emory University (Dunlop et al., 2012, 2017; McGrath et al., 2013). The recruitment procedures, inclusion and exclusion criteria for MDD patients, and parameters MRI data acquisition have been previously described and are detailed in the eMethods. In summary, all MDD patients included in the present study were screened for acute presence of MDD using standardized interview procedures administered by (at minimum) one certified mental health professional. Healthy control subjects were screened (at minimum) for absence of any history of psychiatric illness.

### 2.5. Grouping of primary data cohorts

Participants from the GOBS and Emory cohorts were further screened for subsequent subgrouping based on available clinical features of MDD. We screened for treatment naive MDD, absence of psychiatric comorbidities, first episode and recurrent depression. Details of disease severity, current medication status, and treatment resistance were also noted (where reported) for potential clinical grouping.

### 2.6. Primary data processing and sampling

T1-weighted structural data from both the GOBS and Emory groups were processed and sampled using tools from FSL-VBM procedure (Smith et al., 2004) and Mango software (Mango version 4.1). Details for the generation of gray matter images are described in the eMethods. 6 mm diameter spherical ROIs were used to extract mean gray matter volume values within each node for each subject.

rs-fMRI data, available only in the GOBS groups, was processed and sampled using tools from FSL (Smith et al., 2004). 12 mm diameter spherical ROIs were used to extract time series BOLD values within each node for each subject. Further details are available in the eMethods

### 2.7. SEM measures

SEM models were constructed and assessed using the Analysis of Moment Structures (Amos) version 26.0 program (Arbuckle, 2014). Standardized path coefficients (i.e. values between −1 and 1) were used when testing differences at the level of individual edges within the node-and-edge model. To test for differences in path coefficients between groups, we first conducted a Fisher’s *Z* transformation of standardized path coefficients. Next, *z*-tests between individual path coefficient values across paired groups was performed. Computation of effect size for difference in path coefficients across paired groups preceded by using Cohen’s q (Smallwood et al., 2019).

The root mean square error approximation (RMSEA) was used to asses model goodness of fit for this study. Models attaining an upper limit RMSEA of 0.08 may be considered well-fitting (Li et al., 2016; Hooper et al., 2008), with improved model fit at increasingly smaller values of RMSEA and perfect models being represented by RMSEA of 0.00 (Kenny, 2024; Cangur and Ercan, 2015). ΔRMSEA of 0.01 or greater between two (invariant groups, see below) groups may be considered as a significant model fit difference (Putnick and Bornstein, 2016).

Amos facilitated two additional SEM assessments for the present study: a) modeling autoregressive components in resting-state datasets, and b) testing of model invariance across paired groups for which model fit measures and path coefficients were to be compared. Detailed methods for these SEM best practices are described in the eMethods and only results presented in the main text Section 3.

### 2.8. Meta-SEM fitting

The candidate model was created using the Amos program (Arbuckle, 2014) and fit to several meta-analytic datasets for preliminary assessment of model goodness of fit prior to fitting in primary patient data. The model was first fit in normal activation data from the BrainMap database and in the MDD-specific meta-analytic dataset. The normal activation data was collected from the BrainMap task-activation sector restricted to “healthy subjects” and “normal mapping”. The modeled-activation (MA) maps collected from this sample comprised data from 9359 individual experiments. 9 mm diameter spherical ROIs were used to sample ALE values in all normal subjects’ activations in the BrainMap database. 9 mm spherical ROIs were also used to sample the per-experiment, unified all effects meta-analytic data from the original CBMA. The original CBMA data was subsequently sub-divided into its component data types of voxel-based morphometry decreases (VBM−), voxel-based pathophysiology increases (VBP+), and voxel-based pathophysiology decreases (VBP−) for ROI resampling in the effect-specific meta-data.

### 2.9. Primary-SEM fitting

The candidate model was subsequently fit to the primary structural and rs-fMRI data. Differences in individual path coefficients and RMSEA were compared across paired MDD-patient and control groups.

### 2.10. Voxel-based morphometry of primary data

To assess the mass univariate signal (without a priori regional sampling) of the procured primary datasets, the previously described FSL-VBM (Smith et al., 2004) procedure was carried out to include voxel-wise general linear modeling (GLM) to test for groupwise differences in gray matter volume between patients and controls. The procedure followed the same parameters used in the previous step for groupwise gray matter image creation. Subsequent modifications to the procedure, such as elimination of select scans, were required for some patient-control subgroups (see eTables 3–7 in supplemental content) due to the sensitivity of the voxelwise GLM procedure.

VBM results were thresholded using threshold-free cluster enhancement (TFCE) with 5000 permutations on the modulated, smoothed (2 mm kernel) gray matter images. We tested for both instances of decreased gray matter volume in MDD patients compared to controls (gray matter atrophy in MDD) and increased gray matter volume in patients compared to controls (MDD hypertrophy). Resultant clusters attaining a value of p < 0.05 were considered significant.

To assess the mass univariate signal of VBM processed data with a priori regional sampling, t-tests were performed to test for significant differences in mean gray matter volume for each node (6 mm diameter spherical ROIs) between MDD patients and controls in all clinical subgroups.

These collective methods served to test “strength” of MDD signal in three tiers: mass univariate, (with and without a priori regional sampling), and multivariate (SEM modeling).

## 3. Results

### 3.1. Nodes identified

14 nodes representing distinct brain areas were identified from the top 0.5 % peaks from the unthresholded ALE union map. The node regions included areas of the: subgenual cingulate, left caudate, left hippocampus, an additional area of the left caudate including portions of the left thalamus, left retrosplenial cortex, left middle frontal gyrus, right putamen, left amygdala, left superior temporal gyrus, left uncus, left inferior frontal gyrus, left medial frontal gyrus, left precentral gyrus, and right insula. The nodes and the location of each node’s centroid (Talairach space) are listed in Table 1 in order of decreasing ALE and Z significance from the thresholded ALE union map.

### 3.2. Edges identified

**Fig. 1.**
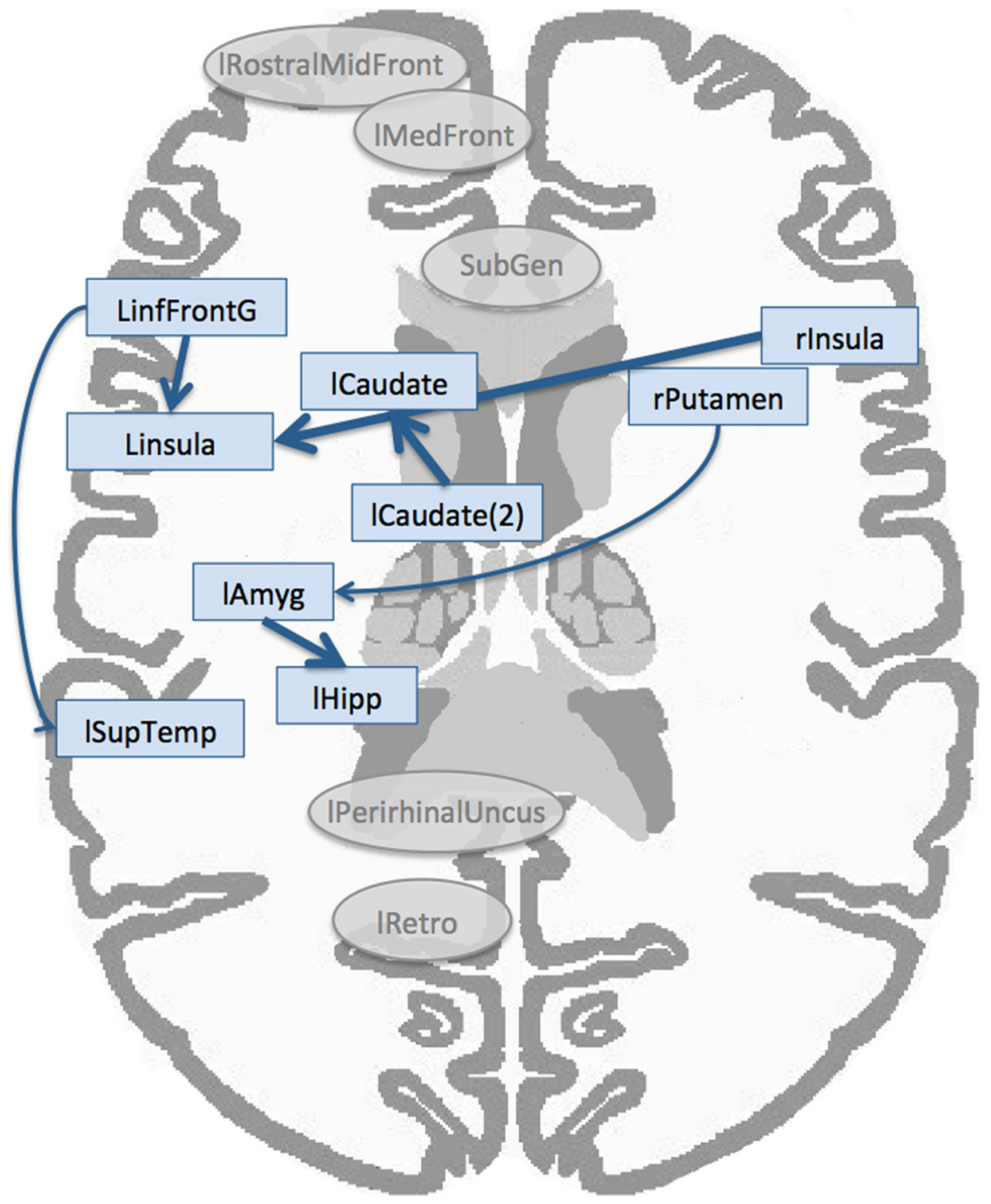
SEM model derived from fMACM modeling. Candidate SEM model consisting of 9 nodes and 6 edges. Note that 5 of 14 originally tested MDD nodes did not demonstrate reliable co-alteration with other MDD nodes as determined through fMACM. Nodes tested, but not included in final model are shown in gray. Model is displayed in approximate anatomical space, axial view, neurological left-right orientation. Abbreviations: LinfFrontG- left inferior frontal gyrus, Linsula- left insula, lSupTemp- left superior temporal gyrus, rInsula-right insula, lAmyg- left amygdala, lHipp- left hippocampus, lCaudate- left caudate, lCaudate(2)- left caudate(2).

6 edges demonstrating a z score above 3.0 were identified through the fMACM procedure. The significant connections spanned between 9 of the total 14 nodes tested through fMACM. The resulting 9-node 6-path model is shown in Fig. 1.

Among the 14 original nodes, a total of 182 node-to-node paths were tested using each node as a seed and checking all potential connections between each seed and the remaining 13 nodes. Nodes tested in the fMACM procedure which did not demonstrate any above threshold connections are shown in Fig. 1 as gray regions with no connecting paths. Unconnected nodes included regions of the subgenual cingulate, left medial frontal gyrus, left middle frontal gyrus, left uncus, and left retrosplenial cortex. Unconnected nodes were not included in the final SEM model. Bi-directional z score values for all 182 paths tested are available in the eFigure 2.

### 3.3. Patient groups identified

Participants from the GOBS cohort were grouped in two tiers: 1) an all patients heterogenous group (***MDDall***) and 2) patient subgroups delineated by clinical category. The clinical categories identified within the GOBS cohort were: a) MDD with psychiatric comorbidity (***MDD***+), b) MDD without psychiatric comorbidity (***MDDonly***), c) recurrent MDD (***MDDrc***), and d) first-episode MDD (***MDDfe***).

Participants from the Emory cohort were grouped via the two original studies for which they were sourced: 1) Emory group 1 included only treatment naive major depression without psychiatric comorbidities, and 2) Emory group 2 included only major depression patients without psychiatric comorbidity and had ceased any form of prior depression treatment immediately prior to the study period. The patient groups, clinical subgroups, and available clinical characteristics (such as psychiatric comorbidities) for each group are detailed in Table 2.

### 3.4. Model fits in Meta-analytic datasets

Meta-SEM goodness of fit and individual path weights were measured in 5 meta-analytic groups total, shown in Table 3. Overall, with the exception of VBP+ , the model demonstrated acceptable goodness of fit across all meta-analytic groups. Exceptional model fit was demonstrated (RMSEA=0.00) in both the VBM− and VBP− groups.

### 3.5. Model fits in Primary datasets

Model fits across the primary structural and functional datasets are shown in Table 3. The only instances in which model goodness of fit distinguished patients from healthy controls (groupwise) was in structural data from the Emory group 1, Emory group 2, and GOBS-MDDonly groups. In these three cases, the model fit moderately (Emory group 2, RMSEA=0.075) to exceptionally (GOBS-MDDonly, RMSEA=0.00) in MDD patients and fit poorly in controls (RMSEA=0.107–0.171)

Model fits were poor across all healthy control subject groups in the structural data RMSEA= 0.091–0.171), and fit poorly in the more heterogeneous patient groups such as the GOBS MDDfe (RMSEA=0.145) and GOBS MDDrc (RMSEA=0.118) patient groups.

Model fits were very good in all resting-state data across all groups, including both patients and controls (RMSEA= 0.037–0.053). As there was not significant distinction between groupwise model fits in the resting-state data, fitting at the per-subject level was not pursued.

### 3.6. Path differences across groups

Detailed results of invariance testing for all paired groups in the present study are detailed in the eResults. Z-tests for significant differences across all paths, p value for comparison, and effect size (Cohen’s q) are provided in eTable 1 (a-g, T1 groups) and eTable 2 (a-e, functional groups) in the supplemental content. Only one path displayed a statistical difference between MDD patients and healthy controls in the MDDrc group within T1 data, the path from the left hippocampus to the left amygdala (z-difference=−4.112, p = 0.000, q=−0.7841).

### 3.7. VBM Results

Voxel-wise whole-brain VBM results from all subgroups are listed in Table 4 (detailed results in supplemental materials). Overall, very few brain regions demonstrating significant difference in gray matter volume (either volume increases or decreases) were identified between patients and healthy controls. In most subgroups tested, no significant regions of MDD-specific gray matter increases or decreases were identified (Emory group 2, GOBS MDD+, GOBS MDDrc). In VBM assessment of Emory group 1 cohort, regions of the bilateral thalamus (left medial dorsal nucleus, right pulvinar) were identified as demonstrating significant volumetric reductions in MDD patients compared to healthy controls.

Results from t-tests for significant gray matter volume differences between MDD patients and controls from a priori regional sampling are shown in eTable 3. Overall, testing for significant differences from the ALE informed brain regions yielded improved results over the previous univariate analysis. 11 brain areas demonstrated significant gray matter volume difference within p < 0.01 between patients and controls in various clinical subgroups. 12 additional brain areas demonstrated significance within p < 0.05 across patients and controls from various clinical subgroups.

## 4. Discussion

Our findings support our overarching hypothesis that meta-SEM procedure would generate a suitable network model for MDD. In support of our first hypothesis, the meta-SEM model demonstrated good model fits in select meta-analytic, primary structural(1a), and primary functional (1b) MRI data. Per our second hypothesis, the meta-SEM model did successfully distinguish MDD patients from healthy controls by demonstrating good model fit in structural patient data and poor model fit in healthy control structural data (2a). However, this finding was not reflected in the functional MRI data (2b), in that the meta-SEM model fit was good in both MDD patients and healthy controls. Due to the failure of the meta-SEM model to distinguish patients from controls in resting-state data, testing for model fit differences at the per-subject level was not performed. Thus our third hypothesis that model fit differences between patients and controls would be observed at the persubject level was not supported. Model fit testing in patient subgroups, grouped by clinical features, supported our fourth hypothesis that model fit would be deteriorated by increased clinical heterogeneity of MDD patients. Finally, findings from our VBM and meta-SEM analysis of the same primary structural MRI datasets supported our fifth hypothesis of improved multivariate, network-based effects over mass-univariate derived effects.

### 4.1. Meta-SEM MDD Model Impact for Future Clinical Investigation

A recent, well powered study (n = 1809) by Winter and colleagues underscores the lack of disease signal in depression from univariate analysis alone (Winter et al., 2022). Winter suggested that in contrast to univariate approaches, the ability of multivariate methods to model complex relationships may yield improved predictive clinical utility in future studies. Winter et al. also posited that a transition in research approach from univariate to multivariate methods may also facilitate a shift from explanatory modeling of disease effects towards purely predictive modeling. In the present study, we are not posing a neuro-connectomic theory of depression with our meta-SEM model, rather, we are allowing a multivariate “signal” to arise from the imaging data. We demonstrated that our multivariate meta-SEM model can distinguish MDD patients from healthy control subjects in structural T1 data. The next layer of analysis within the proposed predictive pipeline would be to search for more clinical correlates in the patient populations by further refining study parameters to include more details on patient symptoms and treatment effects.

The SEM network modeling approach used in the present study also lends itself to association of neuroimaging findings with other disease correlates in future studies. In recent work by Isvoranu and colleagues, a meta-analytic gaussian graphical model (a special case of meta-SEM) approach was used to elucidate closely correlated clusters of PTSD symptoms and unveiled indications towards clustered subpopulations of PTSD symptoms (Isvoranu et al., 2021). Our finding of variable fit of the MDD model in different clinical subgroups echo findings from Isvoranu’s PTSD study. These findings together support Isvoranu’s suggestion that there may be no one disease-specific network to fit all patients, and future work in disease modeling may benefit from focusing on subpopulations within a diagnosis. In the continued search towards predictive modeling of disorders to in turn inform clinical intervention, SEM’s longstanding popularity in psychometrics and with recent success in the Isvoranu study, we recommend the next step in our MDD study to search for symptom correlates with our MDD meta-SEM model.

In summary, despite a previously volatile literature in depression, we have demonstrated that big-data driven signal can be derived from meta-analysis and validated in primary neuroimaging data. Our findings lend support to ongoing neuroimaging research in MDD at the primary study level, with emphasis on meticulous recruitment and study grouping to best inform future work towards a hopeful predictive framework for classifying patients to maximize treatment outcome.

Details of the meta-SEM MDD model derived in the present study follow in the subsequent discussion sections.

### 4.2. Model fit across meta-analytic and primary datasets

SEM model fitting yielded good overall fit measures in most meta-analytic datasets, which justified our moving forward with a candidate model for assessment in primary patient data. Model fit was moderate (RMSEA=0.089, unified all-effects MDD meta-analytic data) to excellent (RMSEA=0.000, VBM and VBP decreases) across most meta-analytic datasets tested, with the exception of VBP increases for which model fit was very poor (RMSEA=0.322). Examination of the residual matrix of this fit indicated high residual covariance between the right putamen and left insula within the VBP+ data. We broadly interpret this result from the VBP+ findings to excessive noise, inflated false positives, or the possibility that these two brain regions are not physiologically impacted by MDD in the same manner as the other brain areas. Per our over-arching recommendations for future work, we suggest that repeated meta-analysis restricted to single-modality imaging studies and sign of observed effect be conducted for validation of this principle.

A key finding from SEM fitting in primary data was the ability of SEM to distinguish patients from controls in structural T1-weighted MRI data, but not reflect this distinction in the SEM analysis of resting-state fMRI data from the same patient groups. Furthermore, this patient-control distinction was only apparent in more clinically homogeneous datasets and was confounded by potential clinical heterogeneity in other datasets. Per our findings, rs-fMRI (or blood oxygen dependent, BOLD, response) simply does not show the same regional effects in MDD as those in structural MRI findings. Though this finding is notable, it is also a limitation in that the procedure followed in the present study is applicable for only groupwise assessments and not yet useful at the persubject level. For future investigation of neuroimaging-based biomarkers or personalized medicine approaches for MDD, models applicable at the individual subject level will be needed.

### 4.3. Covariance paths

Based on robust assessment of model invariance across groups and path-specific *z*-tests (i.e. testing at p < 0.0007 after correction for multiple comparisons and assessment of effect size for each path difference), no paths displayed statistically significant differences or practical effects between patients and healthy controls across any group. This finding can be considered a limitation of the candidate model derived in the present study. Individual (and reliably) altered paths are more likely to serve as potential neuroimaging-based biomarkers rather than whole models, as clinical application of large-scale network models is currently limited.

### 4.4. Effects of Clinical Heterogeneity

Despite successful distinction between MDD patients and healthy controls through SEM fitting in select groups, failure of this distinction in other patient-control groups may be attributed to effects of clinical heterogeneity. Our finding of the best SEM model fits (moderate RMSEA=0.064 to perfect RMSEA=0.000) exclusively in MDD patient populations with no psychiatric comorbidities, would suggest that the presence of psychiatric comorbidities in MDD patients is a contributing factor to varied findings both in the present study and across MDD neuroimaging literature.

A distinct limitation within these (GOBS) groups is that we were not able to test for potential effect of medication status as these data were not available. Many leading MDD experts posit that medication status, especially emphasizing treatment response, is an important feature to account for in neuroimaging studies of depression (Kennedy et al., 2001). As demonstrated in the Emory cohorts included in the present study, medication status of MDD patients including both treatment naive depression and drug washout prior to study participation, is of growing interest in the field. Despite controlling for these features, variability across MDD patients in regards to treatment response has still yet to demonstrate clear relationship to any clinical characteristics (Dunlop et al., 2012, 2017). Per our overarching recommendations for future work, we suggest that the pipeline used in the present study beginning with meta-analytic model creation followed by subsequent fitting in primary data be replicated while controlling for as many clinical features as possible in both meta-analysis and primary samples.

### 4.5. Identified Brain Areas

Numerous brain regions identified in the present study have been previously reported in MDD literature. Regions of the caudate/thalamus (Cash et al., 2019; Krishnan et al., 1992; Kim et al., 2008), insula (Liu et al., 2010; Disner et al., 2011), putamen (Lu et al., 2016; Keedwell et al., 2005), hippocampus (Sheline et al., 1996; Bremner et al., 2000; Campbell and Macqueen et al., 2004; Sheline, 2011; MacQueen and Frodl, 2011; Sampath et al., 2017; MacQueen et al., 2002; Dillon, 2015; Zeng et al., 2012; Rodríguez-Cano et al., 2014), amygdala (Victor et al., 2010; Hamilton et al., 2008), left inferior frontal gyrus (Keedwell et al., 2005; Bremner et al., 2000), left superior temporal gyrus (Ramezani et al., 2014; Takahashi et al., 2010) have all been hypothesized as potential key brain regions in MDD pathophysiology, and as such warrant further study. Of note, not all brain regions identified in the first tier of our analysis demonstrated reliable connections with other brain regions within this set, and thus were not retained for subsequent SEM model fitting. However, this finding does not preclude these brain areas’ potential importance in MDD. This finding simply indicates that these brain areas do not demonstrate reliable covariance with the other areas used in our study. We recommend future network-based investigations of key MDD-impacted brain regions, including brain areas not addressed in the present study.

### 4.6. Multivariate vs Univariate findings

In the case of primary structural MRI data, the SEM fitting provided more meaningful results than the VBM analysis. Whereas VBM comparison of patients to controls identified very little (one group) to no (three groups) significant differences using mass univariate sampling, disease-control comparison using multivariate (SEM) fitting did strongly distinguish groups in 3 of the 7 samples tested. Of note, the 4 of 7 samples which demonstrated poor model fit in both healthy control and patients MDD patients were more clinically heterogeneous. Per this finding of improved signal detection through multivariate (SEM) assessment of MDD neuroimaging findings compared to univariate (VBM) assessment, we posit that MDD-specific neuroimaging features are detectable and are improved by meta-analytically guided *a priori* selection of MDD-features for multivariate assessment.

## 5. Conclusions & recommendations

Per our previous work, we continue to recommend that neuroimaging studies of depression have very strict recruitment criteria and more carefully defined research questions. This would both improve the interpretation of findings at the individual study level and facilitate more meaningful future meta-analytic studies. From the findings of the present work, MDD-specific neuroimaging effects seem to be more evident at the multivariate level, whereas mass univariate effects seem to be weak. Finally, our finding of distinguishing multivariate disease-control features of MDD in primary structural neuroimaging data, however not in primary resting-state functional, data poses notable implications for future studies utilizing the meta-analytic model creation pipeline employed by the present work.

## Supplementary Material

supp

## Figures and Tables

**Table 1:** Top 0.5 % Peaks (Nodes) from Unthresholded ALE Union Map.

Region Name	Label	TAL Coordinate	Peak ALE Score	Peak Z Score
Subgenual Cingulate	SubGen	2, 30, 0	0.02811	4.420
Left Caudate	lCaudate	−12, 6, 16	0.02796	4.402
Left Hippocampus	lHipp	−24, −16, −12	0.02704	4.290
Left Caudate(2)	lCaudate2	−10, −6, 18	0.02471	3.999
Left Retrosplenial Cortex	lRetro	−12, −46, 8	0.02462	3.988
Left Rostral Middle Frontal Gyrus	lRostralMidFront	−34, 54, 6	0.02407	3.918
Right Putamen	rPutamen	26, 6, 16	0.02397	3.905
Left Amygdala	lAmyg	−26, 0, −10	0.02394	3.901
Left Superior Temporal Gyrus	lSupTemp	−50, −26, 6	0.02312	3.795
Left Perirhinal Cortex/Uncus	lPerirhinalUncus	−18, 2, −34	0.02303	3.783
Left Inferior Frontal Gyrus	LinfFrontG	−46, 10, 22	0.02284	3.758
Left Medial Frontal Gyrus	lMedFrontG	−8, 48, −4	0.02221	3.675
Left Insula	Linsula	−44, 12, 6	0.02195	3.640
Right Insula	rInsula	42, 8, 10	0.2115	3.533

**Table 2:** Patient Demographics.

Group	#M/F	Mean Age	Diagnostic Notes	Other details
**Group 1- GOBS (T1 &rs-fMRI)**				
***MDDall** group*				
128 MDD	39/89	40.85	Acutely ill MDD patients	Medication status unknown
128 HC	39/89	41.09	No psychiatric diagnosis	
***MDD+** subgroup*				
26 MDD+	13/13	46.79	MDD as included diagnosis (with or without psychotic features), with additional diagnoses of anxiety, panic, alcohol and substance abuse	Medication status unknown
26 HC	13/13	46.30	No psychiatric diagnosis	
***MDDonly*** *subgroup*				
26 MDDonly	1/25	43.54	MDD as primary diagnosis (without psychotic features), limited anxiety, panic, and/or phobia symptoms (n = 6)	Recurrent MDD n = 12 Single Episode MDD n = 14 Medication Status Unknown
26 HC	1/25	43.92	No psychiatric diagnosis	
***MDDrc** recurrent subgroup*				
46 recurrent MDD	16/30	39.06	Acutely ill MDD patients presently in recurrent episode, majority of subjects additional diagnoses of anxiety, panic, alcohol and substance abuse (n = 34)	Recurrent MDD patients with psychiatric comorbidities n = 33 Recurrent MDD patients without psychiatric comorbidities n = 11 Medication status unknown
46 HC	16/30	39.80	No psychiatric diagnosis	
***MDDfe** first-episode subgroup*				
58 first-episode MDD	17/41	40.81	Acutely ill MDD patients presently in first episode, majority of subjects additional diagnoses of anxiety, panic, alcohol and substance abuse (n = 48)	First-episode MDD patients with psychiatric comorbidities n = 36 First-episode MDD patients without psychiatric comorbidities n = 18 Medication status unknown
58 HC	17/41	41.37	No psychiatric diagnosis	
Group	#M/F	Mean Age	Diagnostic Notes	Other details
**Group 2- Emory (T1 only)**				
*Emory group 1*				
35 MDD	19/16	41.57	All MDD patients had never received previous treatment for any mood disorder through use of antidepressant	Treatment Naive & No psychiatric comorbidities medication or evidence-based psychotherapy.
35 HC	15/20	35.71	No psychiatric diagnosis	
*Emory group 2*				
35 MDD	15/20	41.28	All MDD patients had to be free of antidepressant use within 7 days of the screening visit (5 weeks for fluoxetine); no currently ongoing psychotherapy at the time of screening; and have had no receipt of electroconvulsive (ECT) therapy within 6 months of the screening visit. Patients were also excluded if they had a lifetime history of failure to respond to 6 or more weeks of treatment with escitalopram oxalate (≥10 mg/d) or 4 or more sessions of CBT for depression.	Treatment washout (or treatment naive) & No psychiatric comorbidities Lifetime treatment resistant depression excluded.
35 HC	15/20	35.71	No psychiatric diagnosis	Same 35 HCs used as matches for MDD Emory group 2

**Table 3:** SEM Model Fit & Path Coefficients across datasets.

Data Group	RMSEA	n	rPutamen > lAmyg	LinfFrontG > Linsula	LinfFrontG > lSupTemp	lCaudate2 > lCaudate	rInsula > Linsula	lHipp > lAmyg
***Meta-Analytic Groups***								
All Activations,CON	**0.044** LO90=0.041 HI90=0.047	9359	0.044	0.166	0.061	0.282	0.225	0.177
MDD MA, All Effects	**0.089** LO90=0.049 HI90=0.126	102	0.474	0.018	−0.005	0.221	0.474	−0.035
MDD MA, VBM− only	**0.000** LO90=0.000 HI90=0.089	43	0.732	0.062	0.054	0.272	0.355	−0.040
MDD MA, VBP+ only	**0.322** LO90=0.276 HI90=0.369	47	−0.057	0.377	−0.035	0.997	−0.023	0.019
MDD MA, VBP− only	**0.000** LO90=0.000 HI90=0.000	62	−0.034	0.000	−0.042	0.813	0.956	−0.016
Data Group	RMSEA	n	rPutamen > lAmyg	LinfFrontG > Linsula	LinfFrontG > lSupTemp	lCaudate2 > lCaudate	rInsula > Linsula	lHipp > lAmyg
***T1 Groups***								
Emory Group 1								
MDD	**0.064** LO90=0.00 HI90=0.15	35	0.186	0.161	0.010	0.539	−0.085	−0.135
CON	**0.171** LO90=0.107 HI90=0.234	35	0.264	−0.144	0.159	0.347	0.425	0.027
Emory Group 2								
MDD	**0.075** LO90=0.00 HI90=0.156	35	0.109	0.100	0.147	0.241	0.327	0.164
CON	**0.107** LO90=0.00 HI90=0.179	35	−0.026	−0.229	0.354	0.414	0.496	0.165
GOBS All								
MDD	**0.092** LO90=0.060 HI90=0.124	128	0.021	−0.095	0.038	0.381	0.339	0.146
CON	**0.092** LO90=0.060 HI90=0.125	128	0.089	−0.103	0.126	0.575	0.287	0.285
Data Group	RMSEA	n	rPutamen > lAmyg	LinfFrontG > Linsula	LinfFrontG > lSupTemp	lCaudate2 > lCaudate	rInsula > Linsula	lHipp > lAmyg
***T1 Groups***								
GOBS MDD+								
MDD+	**0.091** LO90=0.000 HI90=0.185	26	−0.327	−0.158	−0.335	0.533	0.200	0.196
CON	**0.157** LO90=0.067 HI90=0.236	26	0.283	−0.352	−0.017	0.366	0.509	−0.021
GOBS MDDonly								
MDDonly	**0.000** LO90=0.000 HI90=0.129	26	0.360	−0.170	0.060	0.381	0.239	−0.182
CON	**0.141** LO90=0.034 HI90=0.222	26	0.085	−0.117	−0.106	0.616	−0.087	0.296
GOBS recurrent MDD								
MDDrc	**0.118** LO90=0.050 HI90=0.176	46	−0.104	−0.220	0.102	0.359	0.476	−0.118
CON	**0.116** LO90=0.048 HI90=0.175	46	0.212	−0.260	0.173	0.606	0.276	0.451
GOBS first-episode MDD								
MDDfe	**0.145** LO90=0.097 HI90=0.192	58	0.010	0.163	0.151	0.505	0.331	−0.31
CON	**0.130** LO90=0.080 HI90=0.179	58	0.232	−0.364	0.143	0.645	0.288	0.433
Data Group	RMSEA	n	rPutamen > lAmyg	LinfFrontG > Linsula	LinfFrontG > lSupTemp	lCaudate2 > lCaudate	rInsula > Linsula	lHipp > lAmyg
***rs-fMRI Groups***								
GOBS All								
MDDall	**0.045** LO90=0.044 HI90=0.046	128	−0.009	0.222	0.084	0.364	0.174	0.122
CON	**0.037** LO90=0.036 HI90=0.038	128	−0.001	0.206	0.089	0.289	0.195	0.129
GOBS MDD+								
MDD+	**0.046** LO90=0.043 HI90=0.048	26	−0.067	0.199	0.062	0.349	0.142	0.152
CON	**0.036** LO90=0.033 HI90=0.038	26	0.018	0.158	0.035	0.242	0.158	0.169
GOBS MDDonly								
MDDonly	**0.046** LO90=0.043 HI90=0.048	26	0.017	0.159	0.054	0.353	0.134	0.003
CON	**0.040** LO90=0.037 HI90=0.042	26	0.012	0.138	0.080	0.385	0.201	0.142
GOBS recurrent MDD								
MDDrc	**0.044** LO90=0.042 HI90=0.046	46	0.023	0.214	0.095	0.314	0.208	0.106
CON	**0.044** LO90=0.043 HI90=0.046	46	0.006	0.167	0.083	0.341	0.186	0.127
GOBS first-episode MDD								
MDDfe	**0.053** LO90=0.051 HI90=0.055	58	−0.011	0.234	0.078	0.389	0.155	0.171
CON	**0.039** LO90=0.037 HI90=0.040	58	0.008	0.215	0.069	0.368	0.228	0.127

**Table 4:** VBM results from groups.

Group	VBM Result(s)
Emory group 1	MDD>CON: No change CON>MDD: Bilateral thalamus (left medial dorsal nucleus, right pulvinar)
Emory group 2	MDD>CON: No change CON>MDD: No change
GOBS all	MDD>CON: artifacts (brain stem) CON>MDD: artifacts (band/”ringing” at back of brain, cerebellum)
GOBS MDD+comorbid	MDD>CON: No change CON>MDD: No change
GOBS MDDonly	MDD>CON: No change CON>MDD: No change
GOBS recurrent	MDD>CON: No change CON>MDD: No change
GOBS first episode	MDD>CON: No change CON>MDD: No change

## Appendix A. Supporting information

Supplementary data associated with this article can be found in the online version at doi:10.1016/j.bosn.2025.04.008.
