# Supplementary material for: Structural and functional covariance architecture of major depressive disorder: A meta-analytic structural equation modeling approach to primary neuroimaging analysis
Source: Brain Organoid Syst Neurosci J. Author manuscript; Available in PMC 2026 Jul 10. (PMC13347272; doi:10.1016/j.bosn.2025.04.008)
Supplement: supp [file NIHMS2181613-supplement-supp.docx]

**Supplementary Online Content**

Gray JP, Price LR, Franklin C, Leonardo CD, Chiang FL, Choi KS, Blangero J, Glahn DC, Mayberg HS, Fox PT. Structural and Functional Covariance Architecture of Major Depressive Disorder: A Meta-Analytic Structural Equation Modeling Approach to Neuroimaging Analysis.

**eMethods 1.** Primary Patient Data Cohorts

**eMethods 2.** FSL-VBM Processing Methods

**eMethods 3.** FSL rs-fMRI Processing Methods

**eMethods 4.** SEM Methods for Modeling Autoregressive Components of rs-fMRI and Invariance Analysis across Paired Groups

**eMethods 5.** Details of Literature Search & Study Selection for Meta-Analysis

**eFigure 1.** SEM Model with Autoregressive Components for rs-fMRI

**eFigure 2.**  Bi-directional fMACM Tests for all Paths (Z-scores)

**eFigure 3.** VBM results

**eResults 1.**  Results from Invariance Testing Across T1-Structural data

**eResults 2**. Results from Invariance Testing Across Functional rs-fMRI data

**eTable 1 (a-g).** Pathwise comparisons across T1 groups

**eTable 2 (a-e).** Pathwise comparisons across functional groups

**eTable 3.** P-values for t-test comparison of a priori regional sampled ROIs for MDD vs healthy controls in all clinical subgroups

**eTable 4.** Clinical details for patients in GOBS MDDall group

**eTable 5.**  Clinical details for patients in GOBS MDD+ group

**eTable 6.**  Clinical details for patients in GOBS MDDonly group

**eTable 7.**  Clinical details for patients in GOBS MDDrc group

**eTable 8.**  Clinical details for patients in GOBS MDDfe group

**eReferences.** Citations for studies included in meta-analysis

**eMethods 1.** Primary Patient Data Cohorts

The GOBS data originates from extended pedigrees of Mexican-American families within the San Antonio, Texas area for which recruitment parameters are previously described ([Olvera et al., 2011](https://www.ncbi.nlm.nih.gov/pmc/articles/PMC3112290/), Glahn et al., 2012). Subjects in this cohort were screened for psychiatric illness using the semi-structured Mini International Psychiatric Interview Plus (MINI-Plus) ([Sheehan et al., 1998](https://www.psychiatrist.com/jcp/article/pages/1998/v59s20/v59s2005.aspx)). Interviewers administering the MINI-Plus had a postgraduate degree in a mental health field or bachelor’s degree with at least 2 years of related experience. A consensus diagnostic process was employed where cases were reviewed by multiple licensed mental health professionals. Within this cohort, 128 subjects meeting criteria for current MDD were identified for use in the present study, and a corresponding 128 approximate age and gender-matched healthy controls (no psychiatric or major medical illness) were identified from the cohort. Subjects were scanned using a 3T Siemens Trio MRI scanner with an 8-channel head coil. Anatomical images were acquired using T1-weighted TurboFlash with the following parameters: TE=3.0ms, TR=2,100ms and TI=785 with a flip angle of 13 degrees. Resting-state functional images were acquired from the whole-brain using gradient-echo echoplanar imaging (EPI) to detect brain oxygen blood flow (BOLD) with TE= 30ms TR=3000ms at a flip angle of 90 degrees (7.5min). For the resting-state scan, subjects were instructed to lie still with their eyes open, allow their mind to wander, and try not to fall asleep.

The Emory cohort was derived from two subgroups, the first from previously published PReDICT study ([Dunlop et al., 2012](https://www.ncbi.nlm.nih.gov/pubmed/22776534)) and the second from a treatment trial conducted at Emory ([McGrath et al., 2013](https://www.ncbi.nlm.nih.gov/pmc/articles/PMC4413467/)). The Emory PReDICT group (hereafter Emory group 1) evaluated adult subjects between the ages of 18-65 for a current, primary diagnosis of non-psychotic major depression through the Structured Clinical Interview for DSM-IV, confirmed by a psychiatrist’s evaluation, and through a score of at least 18 on the 17-item Hamilton Depression Rating Scale (HAM-D). Additionally, patients in Emory group 1 had to have never received prior treatment for a mood disorder through antidepressant medication or evidence-based psychotherapy. Patients were excluded from Emory group 1 if they had a lifetime history of bipolar disorder, psychotic disorder, dementia, or met DSM-IV criteria in the past 12 months for OCD, eating disorder, (illicit) substance dependence, or dissociative disorder. 3D anatomical scans were acquired using a 3-T whole-body MR system (Siemens MAGNETOM TIM Trio) using magnetization-prepared rapid acquisition gradient-echo sequence with TE=3.02ms, TR= 2,600ms, TI=900ms at a flip angle of 8 degrees.

The second Emory group (hereafter Emory group 2) evaluated adult subjects between the ages of 18-60 who were outpatients with a primary diagnosis of MDD as assessed by the Structured Clinical Interview for DSM-IV-TR Axis I Disorders and confirmed by a study psychiatrist. Additionally, patients in Emory group 2 had to have moderate to severe symptoms of depression, defined as a score of 18 or more on the 17-item HAM-D. Patients were excluded from Emory group 2 if they had a current diagnosis of a primary psychiatric disorder other than MDD; a major medical condition that could contribute to depression; comorbid substance abuse within the past 3 months; substance dependence within 12 months prior to the screening visit, use of antidepressants within 7 days of the screening visit (5 weeks for fluoxetine); current psychotherapy at time of screen; receipt of electroconvulsive therapy within 6 months of the screening; and other standard exclusionary criteria for participation in clinical trials. Patients were also excluded if they had a lifetime history of failure to respond to standardized treatments for depression. 3D anatomical scans were acquired using a TIM Trio 3-T whole-body scanner (Siemens) using the following imaging parameters: magnetization-prepared rapid acquisition with gradient echo optimized at TE= 5ms, TR= 35ms and 1mm isotropic resolution. Healthy controls were recruited at Emory University and scanned under the same protocol. Further details for all patient cohorts are available in Table 2 and eTables 3-7 in the supplemental content.

1. Olvera, R.L., Bearden, C.E., Velligan, D.I., et al. (2011). Common Genetic Influences on Depression, Alcohol and Substance Use Disorders in Mexican-American Families. *American Journal of Medical Genetics Part B, Neuropsychiatric Genetics*, 156:561–568
2. Glahn, D.C., Laird, A.R., Ellison-Wright, E., et al. (2008). Meta-Analysis of Gray Matter Anomalies in Schizophrenia: Application of Anatomic Likelihood Estimation and Network Analysis. *Biological Psychiatry*, 64(9):774-781
3. Sheehan, D.V., Lecrubier, Y., Sheehan, K.H., et al. (1998). The Mini-International Neuropsychiatric Interview (M.I.N.I.): The Development and Validation of a Structured Diagnostic Psychiatric Interview for DSM-IV and ICD-10. *The Journal of Clinical Psychiatry*, 59(suppl 20):22-33
4. Dunlop, B.W., Binder, E.B., Cubells, J.F., et al. (2012). Predictors of remission in depression to individual and combined treatments (PReDICT): study protocol for a randomized controlled trial. *Trials*, 13:106
5. McGrath, C.L., Kelley, M.E., Holtzheimer, P.E. III, et al. (2013). Toward a Neuroimaging Treatment Selection Biomarker for Major Depressive Disorder. *JAMA Psychiatry*, 70(8):821-829

**eMethods 2.** FSL-VBM Processing Methods

T1-weighted structural data from both the GOBS and Emory groups were processed and sampled using tools from FSL-VBM procedure and Mango software. First, brain extraction and gray-matter segmentation was performed for all anatomical images. Next, a study-specific gray matter template was generated for the group. Next, the per-subject segmented gray matter images were non-linearly registered to the study specific template. Resultant modulated images with a smoothing kernel of 2mm were then used for gray matter volume sampling. Mango software was used to create a 6mm diameter spherical region of interest (ROI) for each node to be sampled. The centroid of each region was derived from the previous *Node discovery* step, and converted to MNI coordinate space to match the output coordinate space from FSL images. ROIs were overlaid on the modulated, smoothed gray matter images from all subjects gray matter volume sampling. The mean value within each ROI for each subject was collected for use in subsequent SEM fitting.

6mm diameter spherical ROIs were used to extract mean gray matter volume values within each node for each subject. The procedure was performed independently on each paired patient-control subgroup identified in the previous step.

**eMethods 3.** FSL rs-fMRI Processing Methods

rs-fMRI data, available only in the GOBS groups, was processed and sampled using tools from FSL (http://fsl.fmrib.ox.ac.uk/fsl/fslwiki). Preprocessing of subjects data included motion correction (MCFLIRT, [Jenkinson et al., 2002](https://www.sciencedirect.com/science/article/pii/S1053811902911328?via%3Dihub)), brain extraction (BET, [Smith et al., 2002](https://www.ncbi.nlm.nih.gov/pubmed/12391568)), spatial smoothing (5mm full-width half-maximum Gaussian kernel), and high-pass temporal filtering (100s), and registration to standard space using FLIRT (Jenkinson and Smith, 2001). 12mm diameter spherical ROIs were used to extract time series BOLD values within each node for each subject. Times series data for each ROI was extracted for all subjects using FSL’s Featquery tool. This procedure produced 150 time point measurements per ROI per subject.

1. Jenkinson, M., Bannister, P., Brady, J.M., et al. (2002). Improved Optimisation for the Robust and Accurate Linear Registration and Motion Correction of Brain Images. *NeuroImage*, 17(2):825-841
2. Smith S. (2002). Fast robust automated brain extraction. Hum Brain Mapp, 17(3): 143-55.

**eMethods 4.** SEM Methods for Modeling Autoregressive Components of rs-fMRI and Invariance Analysis across Paired Groups

Techniques for modeling autoregressive components for time series resting-state data have been previously described to facilitate ”...a flexible, dynamic approach for simultaneously estimating contemporaneous and lagged relationships between ROIs“ ([Smallwood et al., 2019](https://www.frontiersin.org/articles/10.3389/fnhum.2019.00174/full)). For the present study, a lagged variable (*LAG-A*) was created for each observed ROI (*A*) by offsetting each time point by one. Lagged variables were added to the SEM as observed variables and were loaded onto model components in the following manner: for each connection in the original SEM ROI *A* to ROI *B*, the lagged component of ROI *A* was modeled by loading *LAG-A* to *A*, *LAG-A* to *B*, and *LAG-A* to *LAG-B*. This modeling strategy was used only in tests of primary resting-state data to capture both delayed effects of connections ROI *A* to ROI *B* and account for autocorrelation ([Li et al., 2016](https://www.ncbi.nlm.nih.gov/pmc/articles/PMC4905965/)).

SEM-based invariance analysis, or simply “invariance”, is used in multigroup comparisons of the same SEM to verify that model measurements represent the same model across study groups ([Cheung & Rensvold, 1999](https://www.sciencedirect.com/science/article/pii/S0149206399800014)). Testing of invariance across all paired MDD patient and healthy control groups in the present study was facilitated through use of Amos’ strategy for “Simultaneous Analysis for Several Groups”. This procedure first treats all members of two separate groups, such as an MDD group and healthy control group, as a single unified group to perform initial SEM model fitting. Standardized SEM fit measures such as RMSEA, comparative fit index (CFI) and chi-square values of overall model fit for the combined or “unconstrained” model are initially generated. Next, the path coefficients and individual variable variances for each separate group are constrained to be equal to that of the original unconstrained group model. Model fitting is then repeated in the two constrained groups with the “structural weights” and “structural residuals” constrained to the original unconstrained model. Significant improvement in the constrained models via assessment of updated chi-square and p value indicate that potential per-path non-invariance across groups be assessed. Next, each individual path in the group models are constrained to be equal to the value within the unconstrained model. Individual constrained paths and re-testing of model fit is repeated iteratively for each path. Significant improvement in the per-path constrained models via assessment of updated chi-square and p value indicate that a particular path may be non-invariant across groups.

1. Smallwood, R.F., Price, L.R., Campbell, J.L. et al. (2019). Network Alterations in Comorbid Chronic Pain and Opioid Addiction: An Exploratory Approach. *Frontiers in Human Neuroscience*, 13:174
2. Li, K., Laird, A.R., Price, L.R., et al. (2016). Progressive Bidirectional Age-Related Changes in Default Mode Network Effective Connectivity across Six Decades. *Frontiers Aging Neuroscience*, 8:137
3. Cheung, G.W., Rensvold, R.B. (1999). Testing factorial invariance across groups: A reconceptualization and proposed new method. *Journal of Management*, 25(1):1-27

**eMethods 5.** Details of Literature Search & Study Selection

*Literature Search*

A literature search of PubMed, Google Scholar, BrainMap and reference tracing of previous meta-analyses was performed to identify MDD neuroimaging experiments reporting either gray matter atrophy, or increased resting state function, or decreased resting state function compared to healthy control subjects. MDD related hypertrophy, a rare phenomenon occasionally reported in remitted MDD (relative to acute MDD), was not included in this analysis. Voxel-based morphometry (VBM) studies and resting state voxel-based pathophysiology (VPB) studies of regional cerebral blood flow (rCBF), regional homogeneity (ReHo), amplitude of low frequency fluctuations (ALFF/fALFF), and regional glucose metabolism were identified using various combinations of the search terms *major depressive disorder, major depression, depression, unipolar depression, VBM, gray matter, rCBF, positron emission tomography (PET), single photon emission computed tomography (SPECT), arterial spin labeling (ASL), ReHo, ALFF/fALFF, glucose metabolism, brain activity, and resting state.* The literature search was completed January 2018. No review protocol was used for the present meta-analysis.

Neuroimaging experiments reporting gray matter atrophy (using MRI) or reporting increased or

decreased resting-state function (using fMRI, PET, or SPECT) in patients with major depressive

disorder (MDD) compared to controls and published between September 1992 and January 2018 were identified by PubMed, Google Scholar, and reference tracing of previous meta-analyses in MDD. Search terms included different combinations of: “voxel-based morphometry”, “VBM”, “gray matter”, “gray matter atrophy”, “gray matter volume”, “gray matter concentration”,

“whole-brain”, “resting-state”, “brain function”, “brain activity”, “regional cerebral blood flow”,

“rCBF”, “regional homogeneity”, “ReHo”, “amplitude of low frequency fluctuations”,

“ALFF/fALFF”, “glucose metabolism”, “arterial spin labeling”, “ASL”, “positron emission

tomography”, “PET”, “FDG-PET”, “O-15 PET”, “single photon emission tomography”,

“SPECT”, “major depressive disorder”, “MDD”, “major depression”, “depression”, and

“unipolar depression”. The BrainMap voxel-based morphometry database was also queried for qualifying experiments demonstrating reduced gray matter volume in MDD patients relative to controls. Parameters for the BrainMap search included: “Experiments: Contrast is: Gray Matter”, “Subjects: Diagnosis is: Major Depressive Disorder (MDD)”, and “Experiments: Observed Changes is: Controls > Patients”. General exclusion criteria included: ROI-based or non voxel-wise whole-brain studies, functional or effective connectivity studies, or experiments which reported MDD patients in remission or any other contrast than acutely ill MDD patients vs. healthy controls.

*Study selection criteria relating to subjects*

MDD patients from included studies were diagnosed using DSM-III (4 studies), DSM-IV (85 studies), or ICD-10 (3 studies) evaluation. Only studies comparing patients in the acute phase of MDD to healthy controls were included. Experiments including remitted subjects (n=2) or any contrast other than MDD vs. healthy controls were excluded (n=5). Studies utilizing dual diagnosis patient populations with other major medical illness (e.g., MDD and hypothyroidism) or psychiatric comorbidities were excluded (n=13). However, we allowed for the inclusion of studies in which partial populations of the patient cohort had comorbidities (e.g., subset of MDD patients with anxiety symptoms) with the criterion that MDD was the primary diagnosis. Studies with strict exclusion criteria for psychiatric comorbidities were flagged for use in subsequent meta-analytic grouping. We allowed for the inclusion of studies that featured patient populations of varying medication status, and flagged those studies which recruited patient populations of specific medication status.

*Study selection criteria relating to technical aspects*

Studies of resting-state VBP included investigations of regional cerebral blood flow (rCBF), regional cerebral glucose metabolism, regional homogeneity (ReHo), and amplitude of low frequency fluctuations (ALFF/fALFF) using imaging methods of positron emission tomography (PET), single photon emission computed tomography (SPECT), and functional magnetic resonance imaging (fMRI). Thirty-six studies investigating functional or effective connectivity were excluded from this meta-analysis based on: regional sampling (n = 20); incompatible patient-group contrasts (n = 9); and, multivariate analyses only, without mass-univarate analyses (n = 7).  Studies investigating changes using task-activation methods were not included in this meta-analysis.

Included studies of gray matter volume utilized voxel-based morphometry methods. Included studies only allowed for those that used voxel-wise whole-brain methods to compare MDD patients to healthy control subjects. Studies using ROI or default mode network-only investigations (n=19) were excluded.

Only studies reporting results as coordinates using standard reference space (Talairach or Montreal Neurological Institute [MNI]) were included; those studies which did not report results in the form of standardized coordinates (n=8) or did not report coordinate system used (n=1) were excluded.  Coordinates were converted to Talairach space for this analysis. To avoid repeated inclusion of the same patient populations we carefully screened studies that pulled from open source or national data repositories and excluded those that reported use of a patient cohort already included in this meta-analysis (n=4).  For multiple studies conducted by the same author, we performed leave-one-out analysis during ALE calculation to assess for potential subsets of repeated patient groups. Studies that may have included subsets of repeated patient groups were excluded from final ALE analysis.

**eFigure 1.** SEM Model with Autoregressive Components for rs-fMRI


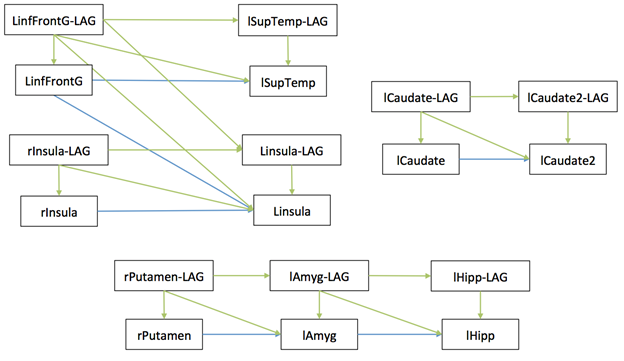


Candidate SEM model with autoregressive lag variables for each observed region of interest (ROI). For each connection in the original SEM ROI *A* to ROI *B*, the lagged component of ROI *A* was modeled by loading *LAG-A* to *A*, *LAG-A* to *B*, and *LAG-A* to *LAG-B*. Note this model visualization is not shown in anatomical space.

e**Figure 2.** Bi-directional fMACM Tests for all Paths (Z-scores)

**
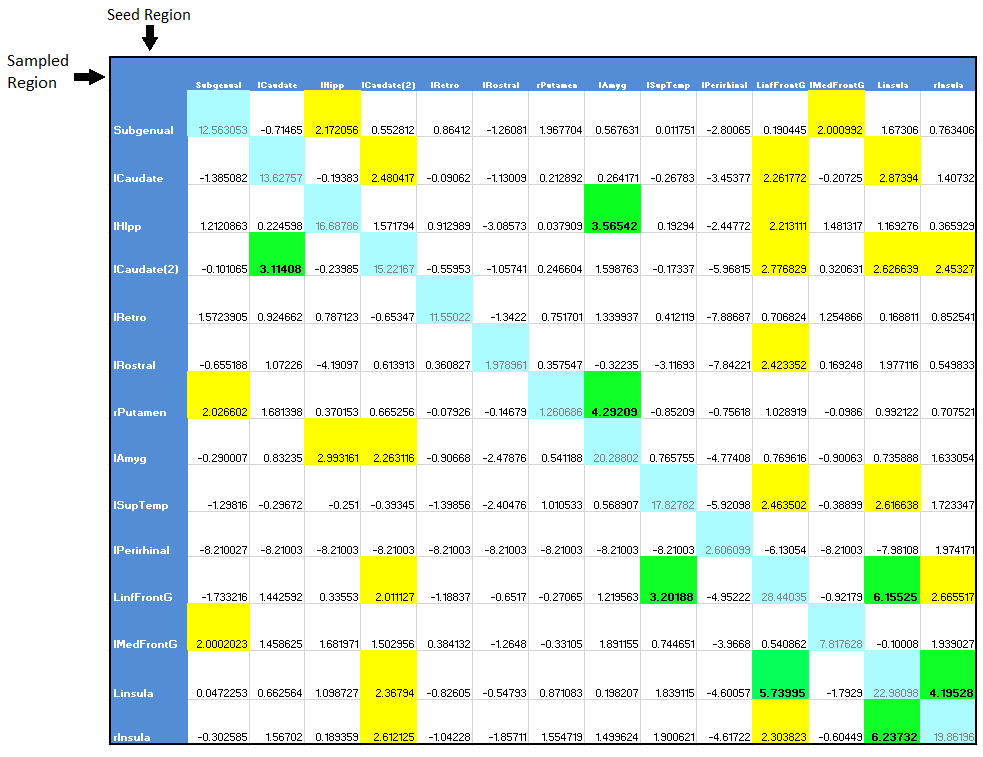
**

All 182 potential connections tested in fMACM. “Self-loop” paths, i.e. z-value for sampling of seed region within its own seed map, are indicated in blue. Paths attaining a z-value of greater than 3.0 are labeled in green, paths attaining a value of greater than 2.0 are labeled in yellow.

**eFigure 3.** VBM Results


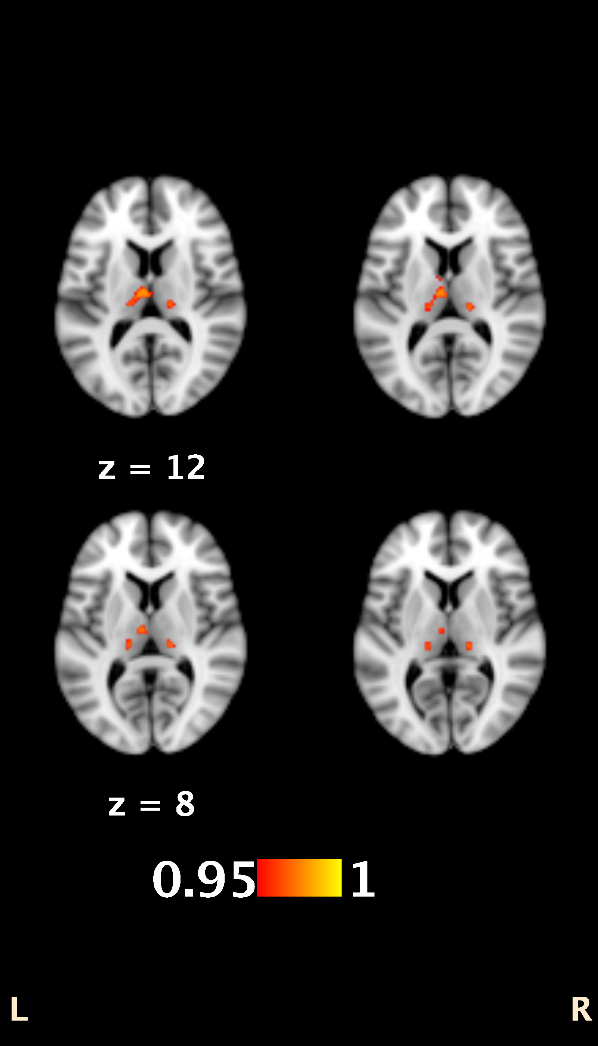


Resultant VBM clusters from Emory Group 1. Clusters included: Thalamus, Medial Dorsal Nucleus (-6,14,12); Thalamus, Pulvinar (16,-24,10); Thalamus, Anterior Nucleus (-8,-2,10); Thalamus, Anterior Nucleus (-6,-4,10).

**eResults 1**. Results from Invariance Testing Across T1-Structural MRI Data

Emory Group 1 T1

Unconstrained, Combined Fit:

Chi-square= 94.119

RMSEA=0.091

CFI=0.0.338

Structural weights constrained Fit:

Chi-square=6.559; p=0.364

Paths constrained Fit:

| **Path Constrained** | **Chi-square of path constrained model** | **P for path constrained model** |
| --- | --- | --- |
| LinfFrontG->Linsula | 1.730 | 0.188 |
| LinfFrontG->lSupTemp | 0.254 | 0.614 |
| rInsula->Linsula | 4.034 | 0.045 |
| rPutamen->lAmyg | 0.646 | 0.422 |
| lAmyg->lHipp | 0.505 | 0.477 |
| lCaudate2->lCaudate | 0.146 | 0.702 |

Emory Group 2 T1

Unconstrained, Combined Fit:

Chi-square= 77.349

RMSEA=0.0.065

CFI=0.515

Structural weights constrained Fit:

Chi-square=3.193; p=0.784

Paths constrained Fit:

| **Path Constrained** | **Chi-square of path constrained model** | **P for path constrained model** |
| --- | --- | --- |
| LinfFrontG>Linsula | 1.948 | 0.163 |
| LinfFrontG->lSupTemp | 0.288 | 0.592 |
| rInsula->Linsula | 0.702 | 0.402 |
| rPutamen->lAmyg | 0.284 | 0.594 |
| lAmyg->lHipp | 0.028 | 0.868 |
| lCaudate2->lCaudate | 0.213 | 0.645 |

GOBS MDDall T1

Unconstrained, Combined Fit:

Chi-square= 124.993

RMSEA=0.065

CFI=0.607

Structural weights constrained Fit:

Chi-square=4.844; p=0.564

Paths constrained Fit:

| **Path Constrained** | **Chi-square of path constrained model** | **P for path constrained model** |
| --- | --- | --- |
| LinfFrontG->Linsula | 0.041 | 0.840 |
| LinfFrontG->lSupTemp | 0.502 | 0.479 |
| rInsula->Linsula | 0.741 | 0.389 |
| rPutamen->lAmyg | 0.251 | 0.617 |
| lAmyg->lHipp | 2.171 | 0.141 |
| lCaudate2->lCaudate | 0.963 | 0.327 |

GOBS MDD+ T1

Unconstrained, Combined Fit:

Chi-square= 84.804

RMSEA=0.0.091

CFI=0.389

Structural weights constrained Fit:

Chi-square=7.259; p=0.298

Paths constrained Fit:

| **Path Constrained** | **Chi-square of path constrained model** | **P for path constrained model** |
| --- | --- | --- |
| LinfFrontG>Linsula | 0.118 | 0.732 |
| LinfFrontG->lSupTemp | 1.234 | 0.267 |
| rInsula->Linsula | 0.505 | 0.477 |
| rPutamen->lAmyg | 4.527 | 0.033 |
| lAmyg->lHipp | 0.410 | 0.522 |
| lCaudate2->lCaudate | 0.574 | 0.449 |

GOBS MDDonly T1

Unconstrained, Combined Fit:

Chi-square= 71.697

RMSEA=0.062

CFI=0.543

Structural weights constrained Fit:

Chi-square=5.153; p=0.524

Paths constrained Fit:

| **Path Constrained** | **Chi-square of path constrained model** | **P for path constrained model** |
| --- | --- | --- |
| LinfFrontG>Linsula | 0.025 | 0.874 |
| LinfFrontG->lSupTemp | 0.323 | 0.570 |
| rInsula->Linsula | 1.399 | 0.237 |
| rPutamen->lAmyg | 0.369 | 0.543 |
| lAmyg->lHipp | 2.875 | 0.090 |
| lCaudate2->lCaudate | 0.186 | 0.666 |

GOBS MDDrc T1

Unconstrained, Combined Fit:

Chi-square= 96.981

RMSEA=0.0.83

CFI=0.569

Structural weights constrained Fit:

Chi-square=13.279; p=0.039

Paths constrained Fit:

| **Path Constrained** | **Chi-square of path constrained model** | **P for path constrained model** |
| --- | --- | --- |
| LinfFrontG>Linsula | 0.328 | 0.567 |
| LinfFrontG->lSupTemp | 0.158 | 0.691 |
| rInsula->Linsula | 1.978 | 0160 |
| rPutamen->lAmyg | 2.422 | 0.120 |
| lAmyg->lHipp | 7.957 | 0.005 |
| lCaudate2->lCaudate | 0.011 | 0.918 |

GOBS MDDfe T1

Unconstrained, Combined Fit:

Chi-square= 124.949

RMSEA=0.0.097

CFI=0.535

Structural weights constrained Fit:

Chi-square=20.976; p=0.002

Paths constrained Fit:

| **Path Constrained** | **Chi-square of path constrained model** | **P for path constrained model** |
| --- | --- | --- |
| LinfFrontG>Linsula | 7.523 | 0.006 |
| LinfFrontG->lSupTemp | 0.210 | 0.647 |
| rInsula->Linsula | 0.546 | 0.460 |
| rPutamen->lAmyg | 1.248 | 0.264 |
| lAmyg->lHipp | 8.163 | 0.004 |
| lCaudate2->lCaudate | 0.008 | 0.930 |

**eResults 2**. Results from Invariance Testing Across Functional rs-fMRI data

GOBS MDDall rs-fMRI

Unconstrained, Combined Fit:

Chi-square= 8538.818

RMSEA=0.029

CFI=0.755

Structural weights constrained Fit:

Chi-square=168.377; p=0.000

Paths constrained Fit:

| **Path Constrained** | **Chi-square of path constrained model** | **P for path constrained model** |
| --- | --- | --- |
| LinfFrontG->Linsula | 2.607 | 0.106 |
| LinfFrontG->lSupTemp | 0.221 | 0.638 |
| rInsula->Linsula | 4.620 | 0.032 |
| rPutamen->lAmyg | 0.630 | 0.427 |
| lAmyg->lHipp | 0.423 | 0.515 |
| lCaudate2->lCaudate | 59.918 | 0.000 |

GOBS MDD+ rs-fMRI

Unconstrained, Combined Fit:

Chi-square= 2056.169

RMSEA=0.030

CFI=0.736

Structural weights constrained Fit:

Chi-square=85.197; p=0.000

Paths constrained Fit:

| **Path Constrained** | **Chi-square of path constrained model** | **P for path constrained model** |
| --- | --- | --- |
| LinfFrontG>Linsula | 7.693 | 0.006 |
| LinfFrontG->lSupTemp | 0.645 | 0.419 |
| rInsula->Linsula | 7.252 | 0.007 |
| rPutamen->lAmyg | 12.258 | 0.000 |
| lAmyg->lHipp | 0.201 | 0.654 |
| lCaudate2->lCaudate | 2.995 | 0.084 |

GOBS MDDonly rs-fMRI

Unconstrained, Combined Fit:

Chi-square= 2056.587

RMSEA=0.030

CFI=0.725

Structural weights constrained Fit:

Chi-square=126.437; p=0.000

Paths constrained Fit:

| **Path Constrained** | **Chi-square of path constrained model** | **P for path constrained model** |
| --- | --- | --- |
| LinfFrontG>Linsula | 0.928 | 0.335 |
| LinfFrontG->lSupTemp | 1.274 | 0.259 |
| rInsula->Linsula | 9.063 | 0.003 |
| rPutamen->lAmyg | 0.061 | 0.805 |
| lAmyg->lHipp | 38.236 | 0.000 |
| lCaudate2->lCaudate | 2.413 | 0.120 |

GOBS MDDrc rs-fMRI

Unconstrained, Combined Fit:

Chi-square= 3639.398

RMSEA=0.031

CFI=0.727

Structural weights constrained Fit:

Chi-square=49.123; p=0.006

Paths constrained Fit:

| **Path Constrained** | **Chi-square of path constrained model** | **P for path constrained model** |
| --- | --- | --- |
| LinfFrontG>Linsula | 7.711 | 0.005 |
| LinfFrontG->lSupTemp | 0.488 | 0.485 |
| rInsula->Linsula | 1.715 | 0.190 |
| rPutamen->lAmyg | 1.021 | 0.312 |
| lAmyg->lHipp | 1.629 | 0.202 |
| lCaudate2->lCaudate | 2.764 | 0.096 |

GOBS MDDfe rs-fMRI

Unconstrained, Combined Fit:

Chi-square= 4971.869

RMSEA=0.033

CFI=0.740

Structural weights constrained Fit:

Chi-square=107.150; p=0.000

Paths constrained Fit:

| **Path Constrained** | **Chi-square of path constrained model** | **P for path constrained model** |
| --- | --- | --- |
| LinfFrontG>Linsula | 1.788 | 0.181 |
| LinfFrontG->lSupTemp | 0.329 | 0.567 |
| rInsula->Linsula | 25.297 | 0.000 |
| rPutamen->lAmyg | 1.639 | 0.200 |
| lAmyg->lHipp | 8.370 | 0.004 |
| lCaudate2->lCaudate | 2.275 | 0.131 |

**eTable 1 (a-g).** Pathwise Comparisons across T1 Groups

**1a. Emory Group 1 (n=35/35)**

**Patients Controls**

| **Path** |  |  | **Path Est.** | **HI 90** | **LO 90** | **p** | **Z'** | **Path Est.** | **HI 90** | **LO 90** | **p** | **Z'** | **z** | **p** | **q** |
| --- | --- | --- | --- | --- | --- | --- | --- | --- | --- | --- | --- | --- | --- | --- | --- |
| **rPutamen** | **>** | **lAmyg** | **0.186** | **0.512** | **-0.134** | **0.319** | **0.1882** | **0.264** | **0.537** | **-0.091** | **0.279** | **0.2704** | **-0.3289** | **0.7414** | **-0.0822** |
| **LinfFrontG** | **>** | **Linsula** | **0.161** | **0.448** | **-0.100** | **0.226** | **0.1624** | **-0.144** | **0.158** | **-0.331** | **0.400** | **-0.1450** | **1.2297** | **0.2187** | **0.3074** |
| **LinfFrontG** | **>** | **LSupTemp** | **0.01** | **0.301** | **-0.248** | **0.813** | **0.0100** | **0.159** | **0.472** | **-0.185** | **0.534** | **0.1604** | **-0.6014** | **0.5485** | **-0.1504** |
| **lCaudate2** | **>** | **lCaudate** | **0.539** | **0.726** | **0.360** | **0.002** | **0.6027** | **0.347** | **0.489** | **0.164** | **0.011** | **0.3620** | **0.9629** | **0.3371** | **0.2407** |
| **rInsula** | **>** | **Linsula** | **-0.085** | **0.136** | **-0.361** | **0.517** | **-0.0852** | **0.425** | **0.572** | **0.201** | **0.013** | **0.4538** | **-2.1559** | **0.0308** | **-0.5390** |
| **lHipp** | **>** | **lAmyg** | **-0.135** | **0.199** | **-0.415** | **0.397** | **-0.1358** | **0.027** | **0.303** | **-0.239** | **0.877** | **0.0270** | **-0.6513** | **0.5157** | **-0.1628** |

**1b. Emory Group 2 (n=35/35)**

**Patients Controls**

| **Path** |  |  | **Path Est.** | **HI 90** | **LO 90** | **p** | **Z'** | **Path Est.** | **HI 90** | **LO 90** | **p** | **Z'** | **z** | **p** | **q** |
| --- | --- | --- | --- | --- | --- | --- | --- | --- | --- | --- | --- | --- | --- | --- | --- |
| **rPutamen** | **>** | **lAmyg** | **0.109** | **0.448** | **-0.187** | **0.568** | **0.1094** | **-0.026** | **0.268** | **-0.277** | **0.959** | **-0.0260** | **0.5418** | **0.5892** | **0.1354** |
| **LinfFrontG** | **>** | **Linsula** | **0.1** | **0.407** | **-0.246** | **0.539** | **0.1003** | **-0.229** | **-0.042** | **-0.440** | **0.056** | **-0.2331** | **1.3339** | **0.1835** | **0.3335** |
| **LinfFrontG** | **>** | **LSupTemp** | **0.147** | **0.464** | **-0.113** | **0.334** | **0.1481** | **0.354** | **0.591** | **0.079** | **0.036** | **0.3700** | **-0.8877** | **0.3735** | **-0.2219** |
| **lCaudate2** | **>** | **lCaudate** | **0.241** | **0.475** | **-0.001** | **0.109** | **0.2458** | **0.414** | **0.598** | **0.208** | **0.010** | **0.4404** | **-0.7784** | **0.4354** | **-0.1946** |
| **rInsula** | **>** | **Linsula** | **0.327** | **0.590** | **0.009** | **0.085** | **0.3395** | **0.496** | **0.656** | **0.262** | **0.013** | **0.5440** | **-0.8181** | **0.4122** | **-0.2045** |
| **lHipp** | **>** | **lAmyg** | **0.164** | **0.417** | **-0.119** | **0.304** | **0.1655** | **0.165** | **0.409** | **-0.121** | **0.365** | **0.1665** | **-0.0041** | **1** | **-0.0010** |

**1c. GOBS MDDall (n=128/128)**

**Patients Controls**

| **Path** |  |  | **Path Est.** | **HI 90** | **LO 90** | **p** | **Z'** | **Path Est.** | **HI 90** | **LO 90** | **p** | **Z'** | **z** | **p** | **q** |
| --- | --- | --- | --- | --- | --- | --- | --- | --- | --- | --- | --- | --- | --- | --- | --- |
| **rPutamen** | **>** | **lAmyg** | **0.021** | **0.180** | **-0.148** | **0.847** | **0.0210** | **0.089** | **0.225** | **-0.053** | **0.344** | **0.0892** | **-0.5394** | **0.5892** | **-0.0682** |
| **LinfFrontG** | **>** | **Linsula** | **-0.095** | **0.046** | **-0.200** | **0.309** | **-0.0953** | **-0.103** | **0.051** | **-0.244** | **0.250** | **-0.1034** | **0.0639** | **0.9522** | **0.0081** |
| **LinfFrontG** | **>** | **LSupTemp** | **0.038** | **0.154** | **-0.092** | **0.600** | **0.0380** | **0.126** | **0.263** | **-0.002** | **0.105** | **0.1267** | **-0.7009** | **0.4839** | **-0.0887** |
| **lCaudate2** | **>** | **lCaudate** | **0.381** | **0.489** | **0.234** | **0.009** | **0.4012** | **0.575** | **0.716** | **0.450** | **0.003** | **0.6550** | **-2.0059** | **0.0444** | **-0.2537** |
| **rInsula** | **>** | **Linsula** | **0.339** | **0.462** | **0.211** | **0.007** | **0.3530** | **0.287** | **0.427** | **0.145** | **0.010** | **0.2953** | **0.4559** | **0.6455** | **0.0577** |
| **lHipp** | **>** | **lAmyg** | **0.146** | **0.273** | **-0.029** | **0.141** | **0.1471** | **0.285** | **0.413** | **0.151** | **0.008** | **0.2931** | **-1.1547** | **0.2501** | **-0.1461** |

**1d. GOBS MDD+ (n=26/26)**

**Patients Controls**

| **Path** |  |  | **Path Est.** | **HI 90** | **LO 90** | **p** | **Z'** | **Path Est.** | **HI 90** | **LO 90** | **p** | **Z'** | **z** | **p** | **q** |
| --- | --- | --- | --- | --- | --- | --- | --- | --- | --- | --- | --- | --- | --- | --- | --- |
| **rPutamen** | **>** | **lAmyg** | **-0.327** | **-0.001** | **-0.552** | **0.099** | **-0.3395** | **0.283** | **0.555** | **-0.034** | **0.151** | **0.2909** | **-2.1378** | **0.0324** | **-0.6304** |
| **LinfFrontG** | **>** | **Linsula** | **-0.158** | **0.304** | **-0.622** | **0.539** | **-0.1593** | **-0.352** | **0.006** | **-0.561** | **0.103** | **-0.3677** | **0.7067** | **0.4777** | **0.2084** |
| **LinfFrontG** | **>** | **LSupTemp** | **-0.335** | **0.122** | **-0.651** | **0.196** | **-0.3484** | **-0.017** | **0.332** | **-0.373** | **0.793** | **-0.0170** | **-1.1240** | **0.2627** | **-0.3314** |
| **lCaudate2** | **>** | **lCaudate** | **0.533** | **0.710** | **0.273** | **0.015** | **0.5943** | **0.366** | **0.573** | **-0.049** | **0.163** | **0.3838** | **0.7139** | **0.4777** | **0.2105** |
| **rInsula** | **>** | **Linsula** | **0.2** | **0.408** | **-0.118** | **0.371** | **0.2027** | **0.509** | **0.665** | **0.150** | **0.048** | **0.5614** | **-1.2162** | **0.225** | **-0.3586** |
| **lHipp** | **>** | **lAmyg** | **0.196** | **0.571** | **-0.381** | **0.623** | **0.1986** | **-0.021** | **0.321** | **-0.441** | **0.838** | **-0.0210** | **0.7446** | **0.4593** | **0.2196** |

**1e. GOBS MDDonly (n=26/26)**

**Patients Controls**

| **Path** |  |  | **Path Est.** | **HI 90** | **LO 90** | **p** | **Z'** | **Path Est.** | **HI 90** | **LO 90** | **p** | **Z'** | **z** | **p** | **q** |
| --- | --- | --- | --- | --- | --- | --- | --- | --- | --- | --- | --- | --- | --- | --- | --- |
| **rPutamen** | **>** | **lAmyg** | **0.36** | **0.650** | **-0.081** | **0.143** | **0.3769** | **0.085** | **0.386** | **-0.231** | **0.731** | **0.0852** | **0.9891** | **0.3222** | **0.2917** |
| **LinfFrontG** | **>** | **Linsula** | **-0.17** | **0.144** | **-0.465** | **0.354** | **-0.1717** | **-0.117** | **0.180** | **-0.442** | **0.522** | **-0.1175** | **-0.1836** | **0.8572** | **-0.0541** |
| **LinfFrontG** | **>** | **LSupTemp** | **0.06** | **0.333** | **-0.208** | **0.724** | **0.0601** | **-0.106** | **0.176** | **-0.405** | **0.617** | **-0.1064** | **0.5645** | **0.5755** | **0.1665** |
| **lCaudate2** | **>** | **lCaudate** | **0.381** | **0.591** | **-0.022** | **0.102** | **0.4012** | **0.616** | **0.803** | **0.265** | **0.004** | **0.7185** | **-1.0760** | **0.2801** | **-0.3173** |
| **rInsula** | **>** | **Linsula** | **0.239** | **0.483** | **0.080** | **0.036** | **0.2437** | **-0.087** | **0.300** | **-0.390** | **0.766** | **-0.0872** | **1.1223** | **0.2627** | **0.3309** |
| **lHipp** | **>** | **lAmyg** | **-0.182** | **0.175** | **-0.514** | **0.325** | **-0.1841** | **0.296** | **0.582** | **-0.052** | **0.161** | **0.3051** | **-1.6589** | **0.0969** | **-0.4892** |

**1f. GOBS MDDrc (n=46/46)**

**Patients Controls**

| **Path** |  |  | **Path Est.** | **HI 90** | **LO 90** | **p** | **Z'** | **Path Est.** | **HI 90** | **LO 90** | **p** | **Z'** | **z** | **p** | **q** |
| --- | --- | --- | --- | --- | --- | --- | --- | --- | --- | --- | --- | --- | --- | --- | --- |
| **rPutamen** | **>** | **lAmyg** | **-0.104** | **0.212** | **-0.416** | **0.700** | **-0.1044** | **0.212** | **0.506** | **-0.051** | **0.158** | **0.2153** | **-1.4821** | **0.1389** | **-0.3196** |
| **LinfFrontG** | **>** | **Linsula** | **-0.22** | **0.028** | **-0.410** | **0.145** | **-0.2237** | **-0.26** | **-0.033** | **-0.471** | **0.067** | **-0.2661** | **0.1968** | **0.8415** | **0.0425** |
| **LinfFrontG** | **>** | **LSupTemp** | **0.102** | **0.279** | **-0.088** | **0.343** | **0.1024** | **0.173** | **0.344** | **-0.080** | **0.286** | **0.1748** | **-0.3357** | **0.7339** | **-0.0724** |
| **lCaudate2** | **>** | **lCaudate** | **0.359** | **0.532** | **0.029** | **0.089** | **0.3757** | **0.606** | **0.749** | **0.445** | **0.004** | **0.7026** | **-1.5155** | **0.1285** | **-0.3268** |
| **rInsula** | **>** | **Linsula** | **0.476** | **0.640** | **0.315** | **0.011** | **0.5178** | **0.276** | **0.470** | **0.022** | **0.086** | **0.2833** | **1.0871** | **0.2757** | **0.2345** |
| **lHipp** | **>** | **lAmyg** | **-0.118** | **0.115** | **-0.425** | **0.377** | **-0.1186** | **0.451** | **0.618** | **0.203** | **0.013** | **0.4860** | **-2.8030** | **0.0051** | **-0.6045** |

**1g. GOBS MDDfe (n=58/58)**

**Patients Controls**

| **Path** |  |  | **Path Est.** | **HI 90** | **LO 90** | **p** | **Z'** | **Path Est.** | **HI 90** | **LO 90** | **p** | **Z'** | **z** | **p** | **q** |
| --- | --- | --- | --- | --- | --- | --- | --- | --- | --- | --- | --- | --- | --- | --- | --- |
| **rPutamen** | **>** | **lAmyg** | **0.01** | **0.271** | **-0.221** | **0.922** | **0.0100** | **0.232** | **0.451** | **-0.020** | **0.111** | **0.2363** | **-1.1867** | **0.234** | **-0.2263** |
| **LinfFrontG** | **>** | **Linsula** | **0.163** | **0.377** | **-0.082** | **0.264** | **0.1645** | **-0.364** | **-0.174** | **-0.500** | **0.017** | **-0.3815** | **2.8630** | **0.0042** | **0.5460** |
| **LinfFrontG** | **>** | **LSupTemp** | **0.151** | **0.354** | **-0.029** | **0.184** | **0.1522** | **0.143** | **0.325** | **-0.054** | **0.237** | **0.1440** | **0.0429** | **0.9681** | **0.0082** |
| **lCaudate2** | **>** | **lCaudate** | **0.505** | **0.640** | **0.293** | **0.016** | **0.5560** | **0.645** | **0.753** | **0.525** | **0.003** | **0.7667** | **-1.1049** | **0.2713** | **-0.2107** |
| **rInsula** | **>** | **Linsula** | **0.331** | **0.571** | **0.103** | **0.021** | **0.3440** | **0.288** | **0.448** | **0.055** | **0.051** | **0.2964** | **0.2494** | **0.8026** | **0.0476** |
| **lHipp** | **>** | **lAmyg** | **-0.31** | **0.209** | **-0.304** | **0.826** | **-0.3205** | **0.433** | **0.588** | **0.236** | **0.011** | **0.4636** | **-4.1120** | **0.0000** | **-0.7841** |

**eTable 2 (a-e). Pathwise Comparisons across functional Groups**

**2a. GOBS MDDall (n=19200/19200)**

**Patients Controls**

| **Path** |  |  | **Path Est.** | **HI 90** | **LO 90** | **p** | **Z'** | **Path Est.** | **HI 90** | **LO 90** | **p** | **Z'** | **z** | **p** | **q** |
| --- | --- | --- | --- | --- | --- | --- | --- | --- | --- | --- | --- | --- | --- | --- | --- |
| **rPutamen** | **>** | **lAmyg** | **-0.009** | **0.005** | **-0.021** | **0.195** | **-0.0090** | **-0.001** | **0.011** | **-0.011** | **0.932** | **-0.0010** | **-0.7838** | **0.4354** | **-0.0080** |
| **LinfFrontG** | **>** | **Linsula** | **0.222** | **0.234** | **0.209** | **0.007** | **0.2258** | **0.206** | **0.218** | **0.193** | **0.008** | **0.2090** | **1.6428** | **0.1010** | **0.0168** |
| **LinfFrontG** | **>** | **LSupTemp** | **0.084** | **0.100** | **0.074** | **0.004** | **0.0842** | **0.089** | **0.103** | **0.077** | **0.007** | **0.0892** | **-0.4936** | **0.6241** | **-0.0050** |
| **lCaudate2** | **>** | **lCaudate** | **0.364** | **0.376** | **0.351** | **0.005** | **0.3815** | **0.289** | **0.304** | **0.277** | **0.003** | **0.2975** | **8.2310** | **0.0000** | **0.0840** |
| **rInsula** | **>** | **Linsula** | **0.174** | **0.184** | **0.161** | **0.011** | **0.1758** | **0.195** | **0.207** | **0.183** | **0.009** | **0.1975** | **-2.1300** | **0.0332** | **-0.0217** |
| **lHipp** | **>** | **lAmyg** | **0.122** | **0.136** | **0.108** | **0.011** | **0.1226** | **0.129** | **0.143** | **0.117** | **0.005** | **0.1297** | **-0.6968** | **0.4839** | **-0.0071** |

**2b. GOBS MDD+ (n=3900/3900)**

**Patients Controls**

| **Path** |  |  | **Path Est.** | **HI 90** | **LO 90** | **p** | **Z'** | **Path Est.** | **HI 90** | **LO 90** | **p** | **Z'** | **z** | **p** | **q** |
| --- | --- | --- | --- | --- | --- | --- | --- | --- | --- | --- | --- | --- | --- | --- | --- |
| **rPutamen** | **>** | **lAmyg** | **-0.067** | **-0.044** | **-0.091** | **0.004** | **-0.0671** | **0.018** | **0.045** | **-0.007** | **0.249** | **0.0180** | **-3.7566** | **0.0020** | **-0.0851** |
| **LinfFrontG** | **>** | **Linsula** | **0.199** | **0.223** | **0.166** | **0.011** | **0.2017** | **0.158** | **0.188** | **0.132** | **0.005** | **0.1593** | **1.8697** | **0.0615** | **0.0424** |
| **LinfFrontG** | **>** | **LSupTemp** | **0.062** | **0.085** | **0.033** | **0.014** | **0.0621** | **0.035** | **0.065** | **0.009** | **0.048** | **0.0350** | **1.1947** | **0.2340** | **0.0271** |
| **lCaudate2** | **>** | **lCaudate** | **0.349** | **0.377** | **0.319** | **0.004** | **0.3643** | **0.242** | **0.277** | **0.219** | **0.006** | **0.2469** | **5.1826** | **0.0000** | **0.1174** |
| **rInsula** | **>** | **Linsula** | **0.142** | **0.176** | **0.117** | **0.003** | **0.1430** | **0.158** | **0.187** | **0.132** | **0.011** | **0.1593** | **-0.7225** | **0.4715** | **-0.0164** |
| **lHipp** | **>** | **lAmyg** | **0.152** | **0.180** | **0.125** | **0.008** | **0.1532** | **0.169** | **0.197** | **0.140** | **0.007** | **0.1706** | **-0.7703** | **0.4413** | **-0.0174** |

**2c. GOB MDDonly (n=3900/3900)**

**Patients Controls**

| **Path** |  |  | **Path Est.** | **HI 90** | **LO 90** | **p** | **Z'** | **Path Est.** | **HI 90** | **LO 90** | **p** | **Z'** | **z** | **p** | **q** |
| --- | --- | --- | --- | --- | --- | --- | --- | --- | --- | --- | --- | --- | --- | --- | --- |
| **rPutamen** | **>** | **lAmyg** | **0.017** | **0.044** | **-0.007** | **0.225** | **0.0170** | **0.012** | **0.035** | **-0.019** | **0.572** | **0.0120** | **0.2208** | **0.8259** | **0.0050** |
| **LinfFrontG** | **>** | **Linsula** | **0.159** | **0.184** | **0.120** | **0.014** | **0.1604** | **0.138** | **0.167** | **0.113** | **0.006** | **0.1389** | **0.9479** | **0.3421** | **0.0215** |
| **LinfFrontG** | **>** | **LSupTemp** | **0.054** | **0.088** | **0.028** | **0.005** | **0.0541** | **0.08** | **0.103** | **0.057** | **0.017** | **0.0802** | **-1.1529** | **0.2501** | **-0.0261** |
| **lCaudate2** | **>** | **lCaudate** | **0.353** | **0.376** | **0.321** | **0.011** | **0.3689** | **0.385** | **0.413** | **0.361** | **0.011** | **0.4059** | **-1.6354** | **0.1010** | **-0.0370** |
| **rInsula** | **>** | **Linsula** | **0.134** | **0.162** | **0.108** | **0.006** | **0.1348** | **0.201** | **0.228** | **0.171** | **0.008** | **0.2038** | **-3.0442** | **0.0024** | **-0.0690** |
| **lHipp** | **>** | **lAmyg** | **0.003** | **0.038** | **-0.023** | **0.757** | **0.0030** | **0.142** | **0.176** | **0.118** | **0.004** | **0.1430** | **-6.1784** | **0.0000** | **-0.1400** |

**2d. GOB MDDrc (n=6900/6900)**

**Patients Controls**

| **Path** |  |  | **Path Est.** | **HI 90** | **LO 90** | **p** | **Z'** | **Path Est.** | **HI 90** | **LO 90** | **p** | **Z'** | **z** | **p** | **q** |
| --- | --- | --- | --- | --- | --- | --- | --- | --- | --- | --- | --- | --- | --- | --- | --- |
| **rPutamen** | **>** | **lAmyg** | **0.023** | **0.041** | **0.000** | **0.101** | **0.0230** | **0.006** | **0.029** | **-0.013** | **0.649** | **0.0060** | **0.9985** | **0.3173** | **0.0170** |
| **LinfFrontG** | **>** | **Linsula** | **0.214** | **0.236** | **0.194** | **0.007** | **0.2174** | **0.167** | **0.186** | **0.146** | **0.014** | **0.1686** | **2.8646** | **0.0042** | **0.0488** |
| **LinfFrontG** | **>** | **LSupTemp** | **0.095** | **0.115** | **0.072** | **0.012** | **0.0953** | **0.083** | **0.103** | **0.060** | **0.012** | **0.0832** | **0.7103** | **0.4777** | **0.0121** |
| **lCaudate2** | **>** | **lCaudate** | **0.314** | **0.338** | **0.294** | **0.007** | **0.3250** | **0.341** | **0.360** | **0.316** | **0.015** | **0.3552** | **-1.7762** | **0.0751** | **-0.0302** |
| **rInsula** | **>** | **Linsula** | **0.208** | **0.233** | **0.192** | **0.004** | **0.2111** | **0.186** | **0.206** | **0.166** | **0.010** | **0.1882** | **1.3442** | **0.1802** | **0.0229** |
| **lHipp** | **>** | **lAmyg** | **0.106** | **0.127** | **0.083** | **0.008** | **0.1064** | **0.127** | **0.149** | **0.105** | **0.005** | **0.1277** | **-1.2502** | **0.2113** | **-0.0213** |

**2e. GOB MDDfe (n=8700/8700)**

**Patients Controls**

| **Path** |  |  | **Path Est.** | **HI 90** | **LO 90** | **p** | **Z'** | **Path Est.** | **HI 90** | **LO 90** | **p** | **Z'** | **z** | **p** | **q** |
| --- | --- | --- | --- | --- | --- | --- | --- | --- | --- | --- | --- | --- | --- | --- | --- |
| **rPutamen** | **>** | **lAmyg** | **-0.011** | **0.007** | **-0.034** | **0.271** | **-0.0110** | **0.008** | **0.025** | **-0.010** | **0.506** | **0.0080** | **-1.2530** | **0.2113** | **-0.0190** |
| **LinfFrontG** | **>** | **Linsula** | **0.234** | **0.254** | **0.219** | **0.005** | **0.2384** | **0.215** | **0.236** | **0.196** | **0.007** | **0.2184** | **1.3195** | **0.1868** | **0.0200** |
| **LinfFrontG** | **>** | **LSupTemp** | **0.078** | **0.095** | **0.060** | **0.010** | **0.0782** | **0.069** | **0.086** | **0.044** | **0.018** | **0.0691** | **0.5967** | **0.5485** | **0.0090** |
| **lCaudate2** | **>** | **lCaudate** | **0.389** | **0.407** | **0.369** | **0.011** | **0.4106** | **0.368** | **0.386** | **0.348** | **0.007** | **0.3861** | **1.6165** | **0.1052** | **0.0245** |
| **rInsula** | **>** | **Linsula** | **0.155** | **0.172** | **0.135** | **0.012** | **0.1563** | **0.228** | **0.246** | **0.213** | **0.005** | **0.2321** | **-4.9998** | **0.0000** | **-0.0758** |
| **lHipp** | **>** | **lAmyg** | **0.171** | **0.196** | **0.155** | **0.005** | **0.1727** | **0.127** | **0.149** | **0.111** | **0.006** | **0.1277** | **2.9679** | **0.0030** | **0.0450** |

**eTable 3.** P-values for t-test comparison of a priori regional sampled ROIs for MDD vs healthy controls in all clinical subgroups

|  | **Emory Group 1** |  |  |  |  |  |  |  |
| --- | --- | --- | --- | --- | --- | --- | --- | --- |
| lAmyg | lCaudate | lCaudate(2) | lHipp | LinfFrontG | Linsula | lSupTemp | rInsula | rPutamen |
| 0.2970 | 0.0011 | 0.0018 | 0.0781 | 0.0708 | 0.3877 | 0.0001 | 0.0129 | 0.1556 |
|  | **Emory Group 2** |  |  |  |  |  |  |  |
| lAmyg | lCaudate | lCaudate(2) | lHipp | LinfFrontG | Linsula | lSupTemp | rInsula | rPutamen |
| 0.0725 | 0.0560 | 0.2196 | 0.0158 | 0.0183 | 0.4008 | 0.0603 | 0.4548 | 0.1907 |
|  | **GOBS MDDall** |  |  |  |  |  |  |  |
| lAmyg | lCaudate | lCaudate(2) | lHipp | LinfFrontG | Linsula | lSupTemp | rInsula | rPutamen |
| 0.0000 | 0.0169 | 0.3075 | 0.1998 | 0.2603 | 0.2431 | 0.0000 | 0.0995 | 0.3993 |
|  | **GOBS MDDonly** |  |  |  |  |  |  |  |
| lAmyg | lCaudate | lCaudate(2) | lHipp | LinfFrontG | Linsula | lSupTemp | rInsula | rPutamen |
| 0.0338 | 0.2295 | 0.3943 | 0.1110 | 0.2436 | 0.4596 | 0.0017 | 0.4754 | 0.1334 |
|  | **GOBS MDD+** |  |  |  |  |  |  |  |
| lAmyg | lCaudate | lCaudate(2) | lHipp | LinfFrontG | Linsula | lSupTemp | rInsula | rPutamen |
| 0.0207 | 0.2426 | 0.0096 | 0.0179 | 0.0003 | 0.0684 | 0.1611 | 0.4805 | 0.1042 |
|  | **GOBS MDDrc** |  |  |  |  |  |  |  |
| lAmyg | lCaudate | lCaudate(2) | lHipp | LinfFrontG | Linsula | lSupTemp | rInsula | rPutamen |
| 0.1442 | 0.0362 | 0.0363 | 0.4912 | 0.4142 | 0.4567 | 0.0005 | 0.0257 | 0.4060 |
|  | **GOBS MDDfe** |  |  |  |  |  |  |  |
| lAmyg | lCaudate | lCaudate(2) | lHipp | LinfFrontG | Linsula | lSupTemp | rInsula | rPutamen |
| 0.0139 | 0.0086 | 0.0131 | 0.3803 | 0.2509 | 0.2469 | 0.0003 | 0.4915 | 0.1318 |

P-values highlighted in green indicate t-test significance within p < 0.01

P-values highlighted in orange indicate t-test significance within p <0.05 (but > 0.01)

**eTable 4.** Clinical Details for Patients in GOBS MDDall Group

| SUBJECT | CLINICAL DETAILS |
| --- | --- |
| MDD001 | MDD, recurrent, w/o psychotic features; GAD, current; and low suicide risk, current |
| MDD002 | Major Depressive Disorder, recurrent, without psychotic features |
| MDD003 | MDD, recurrent, w/o psychotic features; low suicide risk, current; and panic d/o, current, w/ agoraphobia |
| MDD004 | MDD, recurrent; panic d/o, current w/o agoraphobia; GAD, current |
| MDD005 | MDD, recurrent, w/ psychotic features; agoraphobia, lifetime |
| MDD006 | Major Depressive Episode, current, w/o psychotic features; panic d/o, lifetime & current; alcohol dependence, lifetime (duration=72 months) |
| MDD007 | MDD, recurrent; low suicide risk, current |
| MDD008 | MDD, recurrent, w/o psychotic features |
| MDD009 | MDD, recurrent, w/o psychotic features; low suicide risk, current; alcohol dependence, lifetime; substance dependence, lifetime (marijuana); and GAD, current |
| MDD010 | MDE, single episode, current, w/o psychotic features; panic d/o lifetime; alcohol dependence, lifetime (duration=12 months); substance abuse, lifetime (marijuana) |
| MDD011 | Major Depressive Disorder, Recurrent, Current, w/o psychotic features; Panic Disorder Current w/o Agoraphobia; Alcohol Abuse, Current & Lifetime (duration=96 months); Substance Abuse, Current (cocaine) |
| MDD012 | MDD, recurrent, lifetime, w/o psychotic features; specific phobia, current and lifetime, situational type (driving); limited symptom attacks, lifetime |
| MDD013 | Major Depressive Disorder, Recurrent, Current, w/o psychotic features; Low Suicide Risk, Current; Agoraphobia, Current w/o Panic Disorder but w/ past history of panic disorder; Social Anxiety Disorder, Current |
| MDD014 | MDD, recurrent, w/o psychotic features; low suicide risk, current; alcohol abuse, lifetime; substance induced psychotic disorder, lifetime (Lamictal) |
| MDD015 | MDE, single chronic episode, current, w/o psychotic features; low suicide risk, current; alcohol dependence, lifetime (duration=72 months) |
| MDD016 | Major Depressive Disorder, recurrent, without psychotic features; low suicide risk, current; OCD, lifetime & current; Alcohol Dependence, lifetime (duration=12 months); specific phobia, lifetime, situational type |
| MDD017 | MDD, recurrent, w/o psychotic features; high suicide risk, current; OCD, lifetime & current; alcohol dependence, lifetime (duration=12 months); GAD, current |
| MDD018 | MDE, current, single episode, without psychotic features |
| MDD019 | MDD, recurrent, current, chronic, w/o psychotic features; Specific Phobia, environmental (Darkness) Current; PTSD, current. |
| MDD020 | MDD, recurrent, w/o psychotic features |
| MDD021 | MDD, current, single episode, w/o psychotic features; specific phobia, natural environment (storms), lifetime |
| MDD022 | MDD, single episode w/ postpartum onset, current, w/o psychotic features; low suicide risk, current; GAD, current |
| MDD023 | MDD, single episode, current, chronic, w/ psychotic features |
| MDD024 | MDD, current, single episode, w/o psychotic features; specific phobia, lifetime & current, situational type (driving) |
| MDD025 | MDD, recurrent, without psychotic features |
| MDD026 | MDD, recurrent, w/o psychotic features; agoraphobia, current, w/o history of panic d/o; specific phobia, current & lifetime, situational type; alcohol dependence, lifetime (duration=144 months) |
| MDD027 | MDD, recurrent, current; Alcohol Dependence, Current; Substance Dependence, Current. |
| MDD028 | MDD, recurrent, current, w/o psychotic features |
| MDD029 | MDD, single episode, current, w/o psychotic features; Agoraphobia, current w/o history of panic disorder |
| MDD030 | Major Depressive Disorder, past & current, recurrent; Suicide Risk Moderate; OCD lifetime & current; Alcohol Dependence, lifetime & current (duration=120);Substance Dependence, lifetime & current |
| MDD031 | MDD, recurrent & current, w/o psychotic features; Suicide Risk moderate; Panic Disorder w/ Agoraphobia, current; PTSD current; Alcohol Dependence, current (duration = 72 months); Substance Dependence, current (cocaine) |
| MDD032 | MDD, recurrent & current, w/o psychotic features; Low suicide risk, current; Panic Disorder w/ Agoraphobia, current; Alcohol Dependence, lifetime (duration = 240 months); Generalized Anxiety Disorder, current. |
| MDD033 | MDD, recurrent, current, w/o psychotic features; Alcohol Dependence, lifetime (duration=156months) in full remission; Polysubstance Dependence, Lifetime (Heroin explored, but also stimulants, hallucinogens, inhalants and other prescription drugs dependence). |
| MDD034 | MDD, recurrent, current. Alcohol Dependence, Lifetime in full remission. |
| MDD035 | MDD, recurrent & current, chronic, w/o psychotic features; Panic Disorder, life, w/o Agoraphobia; Substance Dependence, lifetime (marijuana) |
| MDD036 | MDD, current, chronic, w/o psychotic features; Panic Disorder, current, w/ Agoraphobia; Alcohol Abuse, lifetime (duration = 120 months); Substance Dependence, lifetime (cocaine) |
| MDD037 | MDD, recurrent, current, w/o psychotic features; Panic Disorder, w/o agoraphobia, lifetime. |
| MDD038 | Major Depressive Disorder, single episode, current w/o psychotic features; Alcohol Dependence, lifetime in full remission (duration = 216 months); Cocaine Dependence, lifetime in full remission |
| MDD039 | Major Depress Disorder, past & current, w/o psychotic features; Limited Symptom Panic Attacks, lifetime & current |
| MDD040 | Major Depressive Disorder, past & current, w/o psychotic features; Low Suicide Risk, current; Social Anxiety Disorder, generalized, lifetime & current |
| MDD041 | Major Depressive Disorder, recurrent & current, chronic, w/o psychotic symptoms; Low Suicide Risk, current |
| MDD042 | Major Depressive Disorder, current, single episode, w/out psychotic features |
| MDD043 | MDD, recurrent, current, w/o psych. features; Agoraphobia, lifetime and current, without Panic Disorder; Social Phobia, lifetime and current; Alcohol Dependence, lifetime and current (duration = 72 months) |
| MDD044 | MDD, past & current, w/o psychotic features; Panic Disorder, lifetime & current, w/ Agoraphobia; Alcohol Dependence, lifetime & current (duration = 72 months); Substance Dependence, current (cocaine); Generalized Anxiety Disorder, current |
| MDD045 | Major Depressive Disorder, single episode, current, w/out psychotic features; Panic Disorder, lifetime; Alcohol Dependence, lifetime and current (duration = 144 months); Substance Dependence, lifetime (cocaine) |
| MDD046 | Major Depressive Disorder, lifetime, recurrent, without psychotic features |
| MDD047 | Major Depressive Disorder, lifetime, single episode, without psychotic features |
| MDD048 | MDD, current, chronic, w/o psychotic features; Panic Disorder, current, w/o Agoraphobia; OCD, lifetime & current; GAD, current |
| MDD049 | Major Depressive Disorder, recurrent, current, without psychotic features; Suicide Risk Current, moderate; PTSD, lifetime and current |
| MDD050 | Major Depressive Disorder, current, single episode, chronic; Generalized Anxiety Disorder, current; PTSD, current (per Olvera, 6/10/2009) |
| MDD051 | Major Depressive Disorder, current, recurrent, without psychotic features; Panic Disorder, current, w/ Agoraphobia; Social Anxiety Disorder, current, generalized Anxiety Disorder, lifetime and current |
| MDD052 | Major Depressive Disorder, single episode, current, w/o psychotic features, mild |
| MDD053 | MDD, recurrent, current, w/o psychotic features; Alcohol Dependence, lifetime (duration = 144 months); GAD, current |
| MDD054 | Major Depressive Disorder, Current, Recurrent, without psychotic features; Suicide Risk Current, Low; Panic Disorder, limited symptoms attacks, Lifetime |
| MDD055 | Major Depressive Disorder, current, without psychotic features; Specific Phobia, lifetime and current, situational (heights); PTSD chronic and current |
| MDD056 | Major Depressive Disorder, lifetime/current, recurrent, w/o psychotic features; Low Suicide Risk, current |
| MDD057 | MDD, current, single episode, w/o Psychotic features; Low Suicide Risk, current; Panic Disorder w/ Agoraphobia, current; Alcohol Dependence, lifetime and current (duration = 48 months); GAD, current |
| MDD058 | Major Depressive Disorder, single episode, current, without psychotic features; Panic Disorder, lifetime and current with Agoraphobia; Alcohol Dependence, lifetime (duration = 108 months); Adult AD/HD |
| MDD059 | Major Depressive Disorder, current, recurrent, w/o PF (per Olvera 11/4/09); Alcohol Dependence, lifetime & current (duration = 348 months); Substance Dependence, lifetime (marijuana) |
| MDD060 | MDD, Recurrent & Current, w/o Psych Features; Low suicide risk; OCD, Lifetime & Current; Alcohol Dependence, Lifetime; Hallucinogen Dependence, Lifetime; Amphetamine Dependence, Lifetime; Opioid Dependence, Lifetime and Current; Hallucinogen-Induced Psychotic Disorder, with Hallucinations, Lifetime |
| MDD061 | Major Depressive Disorder, recurrent and current, without psychotic features; Panic Disorder, lifetime and current; Substance Dependence, lifetime (marijuana); Adult ADHD |
| MDD062 | Major Depressive Disorder, current, without psychotic features; Panic Disorder, lifetime and current, without Agoraphobia; Alcohol Dependence, lifetime and current; Substance Dependence, lifetime (crystal meth); GAD current |
| MDD063 | Major Depressive Disorder, lifetime & current, recurrent, w/o psychotic features |
| MDD064 | MDD, NOS, current; MDD, single episode, past, without Psychotic Features; Specific Phobia, Lifetime and Current (snakes) |
| MDD065 | Major Depressive Disorder, current, chronic, without psychotic features; Suicide Risk Current, low; Alcohol Dependence, current (duration = 24 months); Substance Dependence, current (marijuana) |
| MDD066 | MDD, recurrent, current, w/o psychotic features; Anxiety Disorder NOS, current; Specific Phobia, Lifetime and current (roaches); Current Suicide Risk, Low |
| MDD067 | Depression NOS, current; Alcohol dependence, current; polysubstance dependence, current and lifetime; limited symptom attacks, current; suicide risk, low |
| MDD068 | Major Depressive Disorder, single episode, current, w/o psychiatric features; suicide risk high |
| MDD069 | MDD, recurrent, current, w/o psychotic features; Alcohol dependence, current, in partial remission: Marijuana Dependence, lifetime; low suicide risk |
| MDD070 | Depressive Disorder NOS, Current, Recurrent; Alcohol Dependence, lifetime (72 months); Suicide Risk, low, current; Delusional Disorder, current |
| MDD071 | MDD Recurrent Current, without psychotic features |
| MDD072 | MDD, Current, Recurrent, w/o psychotic features; Anxiety, NOS; Alcohol Dependence, Lifetime & Current; Cannabis Dependence, Lifetime & Current |
| MDD073 | MDD, Current, single episode, w/o Psychotic features; low suicide risk, current; alcohol abuse, lifetime; alcohol dependence, current; substance (marijuana) dependence, lifetime and current; delusional disorder, current and lifetime |
| MDD074 | Depression, NOS, Current; Generalized Anxiety Disorder, Current; Agoraphobia, Lifetime & Current; Social Phobia, Lifetime & Current, generalized; Cannabis Dependence, Lifetime & Current; PTSD, Lifetime; |
| MDD075 | Major Depressive Disorder, Current & Recurrent, w/o Psychotic Features |
| MDD076 | Major Depressive Disorder, Current & Recurrent, w/o Psychotic Features; Social Phobia, Lifetime & Current; Alcohol Dependence, Lifetime; PTSD, Lifetime; |
| MDD077 | MDD, current & recurrent, with psychotic features; Agoraphobia, lifetime & current; Specific Phobia, lifetime & current (driving); Cannabis Abuse, current (10 months); Generalized Anxiety Disorder, Current; Low suicide risk, current (not an Axis I disorder); |
| MDD078 | Major Depressive Disorder, Recurrent & Current; Social Phobia, Lifetime & Current; Alcohol dependence, Lifetime; Generalized Anxiety Disorder, Current; Limited Symptom Panic Attacks (not an axis I disorder); |
| MDD079 | Major Depressive Disorder, current, single episode; agoraphobia, lifetime and current |
| MDD080 | MDD, recurrent, current w/o psychotic features; Social Phobia, lifetime & current |
| MDD081 | Major Depressive Disorder, current and recurrent; agoraphobia, lifetime; social phobia, lifetime; Xanax dependence, lifetime; marijuana abuse, lifetime; cocaine dependence, lifetime |
| MDD082 | Major Depressive Disorder, single episode, current, w/o psychotic features; Low Suicide Risk, current; Generalized Anxiety Disorder, current; Psychotic Disorder NOS, lifetime & current |
| MDD083 | Major Depressive Disorder, current, recurrent; Agoraphobia, lifetime |
| MDD084 | Major Depressive Disorder, recurrent; Marijuana Abuse, current and lifetime |
| MDD085 | Major Depressive Disorder (current); High suicide risk (current); Marijuana dependence (lifetime and current); Alcohol dependence (lifetime and current); O.C.D. (current); Social phobia (current and lifetime); Panic disorder (current and lifetime) |
| MDD086 | Major Depressive Disorder (current); Marijuana dependence (current, lifetime) |
| MDD087 | Major Depressive Disorder(current); low suicide risk (current); Alcohol dependence (lifetime) |
| MDD088 | Major Depressive Disorder, single episode, current, w/o psychotic features; Alcohol Dependence, lifetime & current; Substance Dependence, lifetime & current (cocaine); Substance Dependence, lifetime & current (marijuana); Generalized Anxiety Disorder, current |
| MDD089 | Major Depressive Disorder, Single Episode, Current w/o psychotic features; Specific Phobia, Lifetime & Current (driving); |
| MDD090 | Major Depressive Disorder, single episode, current w/o psychotic features; Moderate Suicide Risk, current |
| MDD091 | Major Depressive Disorder, single episode, current w/o psychotic features |
| MDD092 | Major Depressive Disorder, single episode, past w/o psychotic features; Panic Disorder, lifetime w/o Agoraphobia; Alcohol Dependence, lifetime & current |
| MDD093 | Major Depressive Disorder, Recurrent & Current, w/o Psychotic Features; Panic Disorder, Lifetime & Current, w/ Agoraphobia; Substance Dependence, Lifetime (Xanax); Cannabis Abuse, Lifetime; |
| MDD094 | Major Depressive Disorder, recurrent & current w/o psychotic features; Substance Dependence, lifetime (cocaine); Substance Dependence, lifetime (heroin) |
| MDD095 | Major Depressive Disorder, single episode, past w/o psychotic features; Panic Disorder, lifetime & current w/o Agoraphobia; Social Phobia, lifetime & current; Alcohol Abuse, lifetime; Obsessive-Compulsive Disorder, lifetime & current |
| MDD096 | Major Depressive Disorder, Recurrent & Current w/o Psychotic Features; |
| MDD097 | Major Depressive Disorder, recurrent, current w/o psychotic features; Posttraumatic Stress Disorder, lifetime; Alcohol Dependence, lifetime; Substance Dependence, lifetime & current (cocaine); Substance Abuse, lifetime & current (marijuana); Low Suicide Risk, current |
| MDD098 | Major Depressive Disorder, Recurrent & Current, w/o Psychotic Features; High Suicide Risk, Current; Agoraphobia, Lifetime & Current, w/o History of Panic Disorder; Social Phobia, Lifetime; Alcohol Dependence, Current (4 months duration); Generalized Anxiety Disorder, Current; |
| MDD099 | Major Depressive Disorder, Single Episode, Current, w/o PF; Alcohol Dependence, Lifetime (duration 312 months); Psychotic Disorder, NOS, Lifetime & Current; Low Suicide Risk, Current; |
| MDD100 | Major Depressive Disorder, Single Episode, Current, w/o PF; |
| MDD101 | Major Depressive Disorder, Current, Past, & Recurrent, w/o Psychotic Features; Low Suicide Risk, Current (not an Axis I); |
| MDD102 | Major Depressive Disorder, Recurrent & Current w/o Psychotic Features; Alcohol Dependence, Lifetime; Low Suicide Risk, Current (not an Axis I); |
| MDD103 | Major Depressive Disorder, Single Episode, Current, w/o Psychotic Features; Low Suicide Risk, Current (not an Axis I); |
| MDD104 | Major Depressive Disorder, Current & Recurrent, w/o Psychotic Features; |
| MDD105 | Major Depressive Disorder, Recurrent & Current, w/o Psychotic Features; Low Suicide Risk, Current (not Axis I); Alcohol Dependence, Lifetime & Current (120 month duration); |
| MDD106 | Major Depressive Disorder, Current, & Recurrent, w/o Psychotic Features; Low Suicide Risk, Current (not axis I); Social Phobia, Lifetime & Current; Specific Phobia, Lifetime & Current (heights); PTSD, Lifetime & Current; |
| MDD107 | Major Depressive Disorder, Recurrent & Current, w/o psychotic features; |
| MDD108 | Major Depressive Disorder, Recurrent & Current, w/o psychotic features; High Suicide Risk, Current (not an axis I); Alcohol Dependence, Lifetime (duration 240 months); Heroin Dependence, Lifetime; |
| MDD109 | Major Depressive Disorder, Recurrent & Current, w/o psychotic features; Low Suicide Risk, Current (not an axis I); Specific Phobia, Lifetime & Current (clowns); PTSD, Lifetime; Alcohol Dependence, Lifetime (duration 96 months); Heroin Dependence, Lifetime; Cocaine Dependence, Lifetime; |
| MDD110 | Major Depressive Disorder, Recurrent, w/o Psychotic Features; Low Suicide Risk, Current (not an Axis I disorder); |
| MDD111 | Major Depressive Disorder, Recurrent & Current, w/o Psychotic Features; Alcohol Dependence, Lifetime & Current (duration 36 months); Cannabis Abuse, Lifetime & Current; |
| MDD112 | Major Depressive Disorder, Current & Recurrent, w/o PF; High Suicide Risk, Current (not Axis I); Specific Phobia, Lifetime & Current (claustrophobia); Substance Dependence, Lifetime (heroin); Substance Abuse, Lifetime (marijuana); Childhood ADHD; |
| MDD113 | Major Depressive Disorder, Current, Single Episode, w/o Psychotic Features; Specific Phobia, Lifetime & Current (driving); |
| MDD114 | Major Depressive Disorder, Recurrent & Current, w/o psychotic features; |
| MDD115 | Major Depressive Disorder, Recurrent & Current, w/o Psychotic Features; Low Suicide Risk, Current (not an Axis I disorder); Alcohol Dependence, Lifetime (duration 24 months); Adult ADHD; Psychotic Disorder NOS, Past; |
| MDD116 | Major Depressive Disorder, Recurrent and Current, without Psychotic Features; Alcohol Dependence, Lifetime (duration 2 months); Substance Dependence, Lifetime & Current (Cocaine & Speed); Cannabis Abuse, Lifetime & Current; |
| MDD117 | Depression, NOS, Past; Social Phobia, Lifetime & Current (flying & heights); Alcohol Dependence, Lifetime (120 months); Cannabis Abuse, Lifetime; |
| MDD118 | Major Depressive Disorder, Recurrent & Current, without Psychotic Features; Alcohol Dependence, Lifetime & Current (duration 300 months); Cannabis Abuse, Lifetime; Adult ADHD; Childhood ADHD; |
| MDD119 | Major Depressive Disorder, Current & Recurrent, w/o PF; Moderate Suicide Risk, Current (not Axis I); Substance Dependence, Lifetime (Xanax); Cannabis Abuse, Lifetime; Childhood ADHD; |
| MDD120 | Major Depressive Disorder, Current & Recurrent, without Psychotic Features; Alcohol Dependence, Lifetime & Current (duration 108 months); Panic Disorder, Lifetime & Current, without Agoraphobia; |
| MDD121 | Depression NOS, Past; Major Depressive Disorder, Current, Single Episode, w/o PF; Low Suicide Risk, Current; Alcohol Dependence, Lifetime & Current (duration 120 months); Substance Dependence, Lifetime & Current (heroin & cocaine); Adult ADHD; |
| MDD122 | Major Depressive Disorder, Recurrent & Current; OCD, Lifetime & Current; Heroin Dependence, Lifetime; Specific Phobia, Lifetime & Current (flying & heights); Alcohol Dependence, Lifetime (duration 156 months); |
| MDD123 | Major Depressive Disorder, Current, Single Episode, w/o PF; Moderate Suicide Risk, Current; Social Phobia, Lifetime & Current; Alcohol Dependence, Lifetime (duration 204 months); Substance Dependence, Lifetime (speed); Substance Abuse, Lifetime (marijuana); |
| MDD124 | Major Depressive Disorder, Single Episode, Current, w/o PF; Alcohol Dependence, Lifetime & Current (132 months); |
| MDD125 | Major Depressive Disorder, Single Episode, Current, w/o PF; Social Phobia, Lifetime & Current; Low Suicide Risk, Current (not Axis I); |
| MDD126 | Major Depressive Disorder, Single Episode, Current, w/o psychotic features; Alcohol Abuse, Lifetime (duration 120 months); |
| MDD127 | Major Depressive Disorder, Single Episode, Current, w/o psychotic features; Alcohol Dependence, Lifetime & Current (duration 12 months); Cannabis Dependence, Lifetime; |
| MDD128 | Major Depressive Disorder, Current & Chronic, w/o PF; Moderate Suicide Risk, Current (not Axis I); Panic Disorder, Lifetime & Current, w/o Agoraphobia; Specific Phobia, Situational Type; Psychosis NOS, with Delusion of Persecution; |

**eTable 5.** Clinical Details for Patients in GOBS MDD+ Group

| SUBJECT | CLINICAL DETAILS |
| --- | --- |
| MDD003 | MDD, recurrent, w/o psychotic features; low suicide risk, current; and panic d/o, current, w/ agoraphobia |
| MDD004 | MDD, recurrent; panic d/o, current w/o agoraphobia; GAD, current |
| MDD005 | MDD, recurrent, w/ psychotic features; agoraphobia, lifetime |
| MDD006 | Major Depressive Episode, current, w/o psychotic features; panic d/o, lifetime & current; alcohol dependence, lifetime (duration=72 months) |
| MDD009 | MDD, recurrent, w/o psychotic features; low suicide risk, current; alcohol dependence, lifetime; substance dependence, lifetime (marijuana); and GAD, current |
| MDD010 | MDE, single episode, current, w/o psychotic features; panic d/o lifetime; alcohol dependence, lifetime (duration=12 months); substance abuse, lifetime (marijuana) |
| MDD011 | Major Depressive Disorder, Recurrent, Current, w/o psychotic features; Panic Disorder Current w/o Agoraphobia; Alcohol Abuse, Current & Lifetime (duration=96 months); Substance Abuse, Current (cocaine) |
| MDD012 | MDD, recurrent, lifetime, w/o psychotic features; specific phobia, current and lifetime, situational type (driving); limited symptom attacks, lifetime |
| MDD030 | MDD, recurrent & current, w/o psychotic features; Suicide Risk moderate; Panic Disorder w/ Agoraphobia, current; PTSD current; Alcohol Dependence, current (duration = 72 months); Substance Dependence, current (cocaine) |
| MDD031 | Major Depressive Disorder, past & current, recurrent; Suicide Risk Moderate; OCD lifetime & current; Alcohol Dependence, lifetime & current (duration=120);Substance Dependence, lifetime & current |
| MDD032 | MDD, recurrent & current, w/o psychotic features; Low suicide risk, current; Panic Disorder w/ Agoraphobia, current; Alcohol Dependence, lifetime (duration = 240 months); Generalized Anxiety Disorder, current. |
| MDD033 | MDD, recurrent, current, w/o psychotic features; Alcohol Dependence, lifetime (duration=156months) in full remission; Polysubstance Dependence, Lifetime (Heroin explored, but also stimulants, hallucinogens, inhalants and other prescription drugs dependence). |
| MDD048 | MDD, current, chronic, w/o psychotic features; Panic Disorder, current, w/o Agoraphobia; OCD, lifetime & current; GAD, current |
| MDD049 | Major Depressive Disorder, recurrent, current, without psychotic features; Suicide Risk Current, moderate; PTSD, lifetime and current |
| MDD050 | Major Depressive Disorder, current, single episode, chronic; Generalized Anxiety Disorder, current; PTSD, current (per Olvera, 6/10/2009) |
| MDD057 | MDD, current, single episode, w/o Psychotic features; Low Suicide Risk, current; Panic Disorder w/ Agoraphobia, current; Alcohol Dependence, lifetime and current (duration = 48 months); GAD, current |
| MDD059 | Major Depressive Disorder, current, recurrent, w/o PF (per Olvera 11/4/09); Alcohol Dependence, lifetime & current (duration = 348 months); Substance Dependence, lifetime (marijuana) |
| MDD060 | MDD, Recurrent & Current, w/o Psych Features; Low suicide risk; OCD, Lifetime & Current; Alcohol & Hallucinogen & Amphetamine & Opioid Dep, Lifetime; Hallucinogen-Induced Psych Dis, with Hallucinations, Lifetime |
| MDD062 | Major Depressive Disorder, current, without psychotic features; Panic Disorder, lifetime and current, without Agoraphobia; Alcohol Dependence, lifetime and current; Substance Dependence, lifetime (crystal meth); GAD current |
| MDD069 | MDD, recurrent, current, w/o psychotic features; Alcohol dependence, current, in partial remission: Marijuana Dependence, lifetime; low suicide risk |
| MDD070 | Depressive Disorder NOS, Current, Recurrent; Alcohol Dependence, lifetime (72 months); Suicide Risk, low, current; Delusional Disorder, current |
| MDD073 | MDD, Current, single episode, w/o Psychotic features; low suicide risk, current; alcohol abuse, lifetime; alcohol dependence, current; substance (marijuana) dependence, lifetime and current; delusional disorder, current and lifetime |
| MDD076 | Major Depressive Disorder, Current & Recurrent, w/o Psychotic Features; Social Phobia, Lifetime & Current; Alcohol Dependence, Lifetime; PTSD, Lifetime; |
| MDD082 | Major Depressive Disorder, recurrent & current w/o psychotic features; Substance Dependence, lifetime (cocaine); Substance Dependence, lifetime (heroin) |
| MDD112 | Major Depressive Disorder, Current & Recurrent, w/o PF; High Suicide Risk, Current (not Axis I); Specific Phobia, Lifetime & Current (claustrophobia); Substance Dependence, Lifetime (heroin); Substance Abuse, Lifetime (marijuana); Childhood ADHD; |
| MDD117 | Depression, NOS, Past; Social Phobia, Lifetime & Current (flying & heights); Alcohol Dependence, Lifetime (120 months); Cannabis Abuse, Lifetime; |

**eTable 6.** Clinical Details for Patients in GOBS MDDonly Group

| SUBJECT | CLINICAL DETAILS |
| --- | --- |
| MDD018* (not included in VBM) | MDE, current, single episode, without psychotic features |
| MDD021* (not included in VBM) | MDD, current, single episode, w/o psychotic features; specific phobia, natural environment (storms), lifetime |
| MDD024* (not included in VBM) | MDD, current, single episode, w/o psychotic features; specific phobia, lifetime & current, situational type (driving) |
| MDD028 * (not included in VBM) | MDD, recurrent, current, w/o psychotic features |
| MDD029* (not included in VBM) | MDD, single episode, current, w/o psychotic features; Agoraphobia, current w/o history of panic disorder |
| MDD034 * (not included in VBM) | MDD, recurrent, current. Alcohol Dependence, Lifetime in full remission. |
| MDD041* (not included in VBM) | Major Depressive Disorder, recurrent & current, chronic, w/o psychotic symptoms; Low Suicide Risk, current |
| MDD042* (not included in VBM) | Major Depressive Disorder, current, single episode, w/out psychotic features |
| MDD052* (not included in VBM) | Major Depressive Disorder, single episode, current, w/o psychotic features, mild |
| MDD056* (not included in VBM) | Major Depressive Disorder, lifetime/current, recurrent, w/o psychotic features; Low Suicide Risk, current |
| MDD063 | Major Depressive Disorder, lifetime & current, recurrent, w/o psychotic features |
| MDD064 | MDD, NOS, current; MDD, single episode, past, without Psychotic Features; Specific Phobia, Lifetime and Current (snakes) |
| MDD068 | Major Depressive Disorder, single episode, current, w/o psychiatric features; suicide risk high |
| MDD071 | MDD Recurrent Current, without psychotic features |
| MDD075 | Major Depressive Disorder, Current & Recurrent, w/o Psychotic Features |
| MDD089 | Major Depressive Disorder, Single Episode, Current w/o psychotic features; Specific Phobia, Lifetime & Current (driving); |
| MDD090 | Major Depressive Disorder, single episode, current w/o psychotic features; Moderate Suicide Risk, current |
| MDD091 | Major Depressive Disorder, single episode, current w/o psychotic features |
| MDD096 | Major Depressive Disorder, Recurrent & Current w/o Psychotic Features; |
| MDD100 | Major Depressive Disorder, Single Episode, Current, w/o PF; |
| MDD101 | Major Depressive Disorder, Current, Past, & Recurrent, w/o Psychotic Features; Low Suicide Risk, Current (not an Axis I); |
| MDD103 | Major Depressive Disorder, Single Episode, Current, w/o Psychotic Features; Low Suicide Risk, Current (not an Axis I); |
| MDD104 | Major Depressive Disorder, Current & Recurrent, w/o Psychotic Features; |
| MDD107 | Major Depressive Disorder, Recurrent & Current, w/o psychotic features; |
| MDD113 | Major Depressive Disorder, Current, Single Episode, w/o Psychotic Features; Specific Phobia, Lifetime & Current (driving); |
| MDD114 | Major Depressive Disorder, Recurrent & Current, w/o psychotic features; |

**eTable 7.** Clinical Details for Patients in GOBS MDDrc Group

| SUBJECT | CLINICAL DETAILS |
| --- | --- |
| MDD011 | Major Depressive Disorder, Recurrent, Current, w/o psychotic features; Panic Disorder Current w/o Agoraphobia; Alcohol Abuse, Current & Lifetime (duration=96 months); Substance Abuse, Current (cocaine) |
| MDD013 | Major Depressive Disorder, Recurrent, Current, w/o psychotic features; Low Suicide Risk, Current; Agoraphobia, Current w/o Panic Disorder but w/ past history of panic disorder; Social Anxiety Disorder, Current |
| MDD30 | MDD, recurrent & current, w/o psychotic features; Suicide Risk moderate; Panic Disorder w/ Agoraphobia, current; PTSD current; Alcohol Dependence, current (duration = 72 months); Substance Dependence, current (cocaine) |
| MDD032 | MDD, recurrent & current, w/o psychotic features; Low suicide risk, current; Panic Disorder w/ Agoraphobia, current; Alcohol Dependence, lifetime (duration = 240 months); Generalized Anxiety Disorder, current. |
| MDD034 | MDD, recurrent, current. Alcohol Dependence, Lifetime in full remission. |
| MDD035 | MDD, recurrent & current, chronic, w/o psychotic features; Panic Disorder, life, w/o Agoraphobia; Substance Dependence, lifetime (marijuana) |
| MDD041 | Major Depressive Disorder, recurrent & current, chronic, w/o psychotic symptoms; Low Suicide Risk, current |
| MDD049 | Major Depressive Disorder, recurrent, current, without psychotic features; Suicide Risk Current, moderate; PTSD, lifetime and current |
| MDD051 | Major Depressive Disorder, current, recurrent, without psychotic features; Panic Disorder, current, w/ Agoraphobia; Social Anxiety Disorder, current, generalized Anxiety Disorder, lifetime and current |
| MDD054 | Major Depressive Disorder, Current, Recurrent, without psychotic features; Suicide Risk Current, Low; Panic Disorder, limited symptoms attacks, Lifetime |
| MDD056 | Major Depressive Disorder, lifetime/current, recurrent, w/o psychotic features; Low Suicide Risk, current |
| MDD059 | Major Depressive Disorder, current, recurrent, w/o PF (per Olvera 11/4/09); Alcohol Dependence, lifetime & current (duration = 348 months); Substance Dependence, lifetime (marijuana) |
| MDD060 | MDD, Recurrent & Current, w/o Psych Features; Low suicide risk; OCD, Lifetime & Current; Alcohol Dependence, Lifetime; Hallucinogen Dependence, Lifetime; Amphetamine Dependence, Lifetime; Opioid Dependence, Lifetime and Current; Hallucinogen-Induced Psychotic Disorder, with Hallucinations, Lifetime |
| MDD061 | Major Depressive Disorder, recurrent and current, without psychotic features; Panic Disorder, lifetime and current; Substance Dependence, lifetime (marijuana); Adult ADHD |
| MDD063 | Major Depressive Disorder, lifetime & current, recurrent, w/o psychotic features |
| MDD071 | MDD Recurrent Current, without psychotic features |
| MDD072 | MDD, Current, Recurrent, w/o psychotic features; Anxiety, NOS; Alcohol Dependence, Lifetime & Current; Cannabis Dependence, Lifetime & Current |
| MDD075 | Major Depressive Disorder, Current & Recurrent, w/o Psychotic Features |
| MDD076 | Major Depressive Disorder, Current & Recurrent, w/o Psychotic Features; Social Phobia, Lifetime & Current; Alcohol Dependence, Lifetime; PTSD, Lifetime; |
| MDD077 | MDD, current & recurrent, with psychotic features; Agoraphobia, lifetime & current; Specific Phobia, lifetime & current (driving); Cannabis Abuse, current (10 months); Generalized Anxiety Disorder, Current; Low suicide risk, current (not an Axis I disorder); |
| MDD078 | Major Depressive Disorder, Recurrent & Current; Social Phobia, Lifetime & Current; Alcohol dependence, Lifetime; Generalized Anxiety Disorder, Current; Limited Symptom Panic Attacks (not an axis I disorder); |
| MDD081 | Major Depressive Disorder, current and recurrent; agoraphobia, lifetime; social phobia, lifetime; Xanax dependence, lifetime; marijuana abuse, lifetime; cocaine dependence, lifetime |
| MDD083 | Major Depressive Disorder, current, recurrent; Agoraphobia, lifetime |
| MDD093 | Major Depressive Disorder, Recurrent & Current, w/o Psychotic Features; Panic Disorder, Lifetime & Current, w/ Agoraphobia; Substance Dependence, Lifetime (Xanax); Cannabis Abuse, Lifetime; |
| MDD094 | Major Depressive Disorder, recurrent & current w/o psychotic features; Substance Dependence, lifetime (cocaine); Substance Dependence, lifetime (heroin) |
| MDDrc1 | Major Depressive Disorder, recurrent, current w/o psychotic features; Obsessive-Compulsive Disorder, lifetime & current |
| MDD096 | Major Depressive Disorder, Recurrent & Current w/o Psychotic Features; |
| MDD097 | Major Depressive Disorder, recurrent, current w/o psychotic features; Posttraumatic Stress Disorder, lifetime; Alcohol Dependence, lifetime; Substance Dependence, lifetime & current (cocaine); Substance Abuse, lifetime & current (marijuana); Low Suicide Risk, current |
| MDDrc2 | Major Depressive Disorder, recurrent, current w/o psychotic features |
| MDD098 | Major Depressive Disorder, Recurrent & Current, w/o Psychotic Features; High Suicide Risk, Current; Agoraphobia, Lifetime & Current, w/o History of Panic Disorder; Social Phobia, Lifetime; Alcohol Dependence, Current (4 months duration); Generalized Anxiety Disorder, Current; |
| MDD102 | Major Depressive Disorder, Recurrent & Current w/o Psychotic Features; Alcohol Dependence, Lifetime; Low Suicide Risk, Current (not an Axis I); |
| MDD104 | Major Depressive Disorder, Current & Recurrent, w/o Psychotic Features; |
| MDDrc3 | Major Depressive Disorder, Recurrent & Current, w/o PF; Low Suicide Risk, Current; PTSD, Lifetime; |
| MDD105 | Major Depressive Disorder, Recurrent & Current, w/o Psychotic Features; Low Suicide Risk, Current (not Axis I); Alcohol Dependence, Lifetime & Current (120 month duration); |
| MDD107 | Major Depressive Disorder, Recurrent & Current, w/o psychotic features; |
| MDD108 | Major Depressive Disorder, Recurrent & Current, w/o psychotic features; High Suicide Risk, Current (not an axis I); Alcohol Dependence, Lifetime (duration 240 months); Heroin Dependence, Lifetime; |
| MDD109 | Major Depressive Disorder, Recurrent & Current, w/o psychotic features; Low Suicide Risk, Current (not an axis I); Specific Phobia, Lifetime & Current (clowns); PTSD, Lifetime; Alcohol Dependence, Lifetime (duration 96 months); Heroin Dependence, Lifetime; Cocaine Dependence, Lifetime; |
| MDD111 | Major Depressive Disorder, Recurrent & Current, w/o Psychotic Features; Alcohol Dependence, Lifetime & Current (duration 36 months); Cannabis Abuse, Lifetime & Current; |
| MDD112 | Major Depressive Disorder, Current & Recurrent, w/o PF; High Suicide Risk, Current (not Axis I); Specific Phobia, Lifetime & Current (claustrophobia); Substance Dependence, Lifetime (heroin); Substance Abuse, Lifetime (marijuana); Childhood ADHD; |
| MDD114 | Major Depressive Disorder, Recurrent & Current, w/o psychotic features; |
| MDD115 | Major Depressive Disorder, Recurrent & Current, w/o Psychotic Features; Low Suicide Risk, Current (not an Axis I disorder); Alcohol Dependence, Lifetime (duration 24 months); Adult ADHD; Psychotic Disorder NOS, Past; |
| MDD116 | Major Depressive Disorder, Recurrent and Current, without Psychotic Features; Alcohol Dependence, Lifetime (duration 2 months); Substance Dependence, Lifetime & Current (Cocaine & Speed); Cannabis Abuse, Lifetime & Current; |
| MDD118 | Major Depressive Disorder, Recurrent & Current, without Psychotic Features; Alcohol Dependence, Lifetime & Current (duration 300 months); Cannabis Abuse, Lifetime; Adult ADHD; Childhood ADHD; |
| MDD119 | Major Depressive Disorder, Current & Recurrent, w/o PF; Moderate Suicide Risk, Current (not Axis I); Substance Dependence, Lifetime (Xanax); Cannabis Abuse, Lifetime; Childhood ADHD; |
| MDD120 | Major Depressive Disorder, Current & Recurrent, without Psychotic Features; Alcohol Dependence, Lifetime & Current (duration 108 months); Panic Disorder, Lifetime & Current, without Agoraphobia; |
| MDD122 | Major Depressive Disorder, Recurrent & Current; OCD, Lifetime & Current; Heroin Dependence, Lifetime; Specific Phobia, Lifetime & Current (flying & heights); Alcohol Dependence, Lifetime (duration 156 months); |

**eTable 8.** Clinical details for patients in GOBS MDDfe group

| SUBJECT | CLINICAL DETAILS |
| --- | --- |
| MDD006* (not included in VBM) | Major Depressive Episode, current, w/o psychotic features; panic d/o, lifetime & current; alcohol dependence, lifetime (duration=72 months) |
| MDD010* (not included in VBM) | MDE, single episode, current, w/o psychotic features; panic d/o lifetime; alcohol dependence, lifetime (duration=12 months); substance abuse, lifetime (marijuana) |
| MDD015* (not included in VBM) | MDE, single chronic episode, current, w/o psychotic features; low suicide risk, current; alcohol dependence, lifetime (duration=72 months) |
| MDD018* (not included in VBM) | MDE, current, single episode, without psychotic features |
| MDD019* (not included in VBM) | MDD, recurrent, current, chronic, w/o psychotic features; Specific Phobia, environmental (Darkness) Current; PTSD, current. |
| MDD021* (not included in VBM) | MDD, current, single episode, w/o psychotic features; specific phobia, natural environment (storms), lifetime |
| MDD022* (not included in VBM) | MDD, single episode w/ postpartum onset, current, w/o psychotic features; low suicide risk, current; GAD, current |
| MDD023* (not included in VBM) | MDD, single episode, current, chronic, w/ psychotic features |
| MDD024* (not included in VBM) | MDD, current, single episode, w/o psychotic features; specific phobia, lifetime & current, situational type (driving) |
| MDD027* (not included in VBM) | MDD, recurrent, current; Alcohol Dependence, Current; Substance Dependence, Current. |
| MDD028 | MDD, recurrent, current, w/o psychotic features |
| MDD029 | MDD, single episode, current, w/o psychotic features; Agoraphobia, current w/o history of panic disorder |
| MDD31 | Major Depressive Disorder, past & current, recurrent; Suicide Risk Moderate; OCD lifetime & current; Alcohol Dependence, lifetime & current (duration=120);Substance Dependence, lifetime & current |
| MDD033 | MDD, recurrent, current, w/o psychotic features; Alcohol Dependence, lifetime (duration=156months) in full remission; Polysubstance Dependence, Lifetime (Heroin explored, but also stimulants, hallucinogens, inhalants and other prescription drugs dependence). |
| MDD036 | MDD, current, chronic, w/o psychotic features; Panic Disorder, current, w/ Agoraphobia; Alcohol Abuse, lifetime (duration = 120 months); Substance Dependence, lifetime (cocaine) |
| MDD037 | MDD, recurrent, current, w/o psychotic features; Panic Disorder, w/o agoraphobia, lifetime. |
| MDD038 | Major Depressive Disorder, single episode, current w/o psychotic features; Alcohol Dependence, lifetime in full remission (duration = 216 months); Cocaine Dependence, lifetime in full remission |
| MDD039 | Major Depress Disorder, past & current, w/o psychotic features; Limited Symptom Panic Attacks, lifetime & current |
| MDD040 | Major Depressive Disorder, past & current, w/o psychotic features; Low Suicide Risk, current; Social Anxiety Disorder, generalized, lifetime & current |
| MDD043 | MDD, recurrent, current, w/o psych. features; Agoraphobia, lifetime and current, without Panic Disorder; Social Phobia, lifetime and current; Alcohol Dependence, lifetime and current (duration = 72 months) |
| MDD044 | MDD, past & current, w/o psychotic features; Panic Disorder, lifetime & current, w/ Agoraphobia; Alcohol Dependence, lifetime & current (duration = 72 months); Substance Dependence, current (cocaine); Generalized Anxiety Disorder, current |
| MDD045 | Major Depressive Disorder, single episode, current, w/out psychotic features; Panic Disorder, lifetime; Alcohol Dependence, lifetime and current (duration = 144 months); Substance Dependence, lifetime (cocaine) |
| MDD048 | MDD, current, chronic, w/o psychotic features; Panic Disorder, current, w/o Agoraphobia; OCD, lifetime & current; GAD, current |
| MDD050 | Major Depressive Disorder, current, single episode, chronic; Generalized Anxiety Disorder, current; PTSD, current (per Olvera, 6/10/2009) |
| MDD052 | Major Depressive Disorder, single episode, current, w/o psychotic features, mild |
| MDD053 | MDD, recurrent, current, w/o psychotic features; Alcohol Dependence, lifetime (duration = 144 months); GAD, current |
| MDD055 | Major Depressive Disorder, current, without psychotic features; Specific Phobia, lifetime and current, situational (heights); PTSD chronic and current |
| MDD057 | MDD, current, single episode, w/o Psychotic features; Low Suicide Risk, current; Panic Disorder w/ Agoraphobia, current; Alcohol Dependence, lifetime and current (duration = 48 months); GAD, current |
| MDD058 | Major Depressive Disorder, single episode, current, without psychotic features; Panic Disorder, lifetime and current with Agoraphobia; Alcohol Dependence, lifetime (duration = 108 months); Adult AD/HD |
| MDD062 | Major Depressive Disorder, current, without psychotic features; Panic Disorder, lifetime and current, without Agoraphobia; Alcohol Dependence, lifetime and current; Substance Dependence, lifetime (crystal meth); GAD current |
| MDD066 | MDD, recurrent, current, w/o psychotic features; Anxiety Disorder NOS, current; Specific Phobia, Lifetime and current (roaches); Current Suicide Risk, Low |
| MDD068 | Major Depressive Disorder, single episode, current, w/o psychiatric features; suicide risk high |
| MDDfe1 | Alcohol Dependence, lifetime, in partial remission; MDD, current, w/o psychotic features |
| MDD069 | MDD, recurrent, current, w/o psychotic features; Alcohol dependence, current, in partial remission: Marijuana Dependence, lifetime; low suicide risk |
| MDD073 | MDD, Current, single episode, w/o Psychotic features; low suicide risk, current; alcohol abuse, lifetime; alcohol dependence, current; substance (marijuana) dependence, lifetime and current; delusional disorder, current and lifetime |
| MDD080 | MDD, recurrent, current w/o psychotic features; Social Phobia, lifetime & current |
| MDD082 | Major Depressive Disorder, single episode, current, w/o psychotic features; Low Suicide Risk, current; Generalized Anxiety Disorder, current; Psychotic Disorder NOS, lifetime & current |
| MDD085 | Major Depressive Disorder (current); High suicide risk (current); Marijuana dependence (lifetime and current); Alcohol dependence (lifetime and current); O.C.D. (current); Social phobia (current and lifetime); Panic disorder (current and lifetime) |
| MDD086 | Major Depressive Disorder (current); Marijuana dependence (current, lifetime) |
| MDD087 | Major Depressive Disorder(current); low suicide risk (current); Alcohol dependence (lifetime) |
| MDD088 | Major Depressive Disorder, single episode, current, w/o psychotic features; Alcohol Dependence, lifetime & current; Substance Dependence, lifetime & current (cocaine); Substance Dependence, lifetime & current (marijuana); Generalized Anxiety Disorder, current |
| MDD089 | Major Depressive Disorder, Single Episode, Current w/o psychotic features; Specific Phobia, Lifetime & Current (driving); |
| MDD090 | Major Depressive Disorder, single episode, current w/o psychotic features; Moderate Suicide Risk, current |
| MDD091 | Major Depressive Disorder, single episode, current w/o psychotic features |
| MDDfe2 | Major Depressive Disorder, single episode, current w/o psychotic features |
| MDD099 | Major Depressive Disorder, Single Episode, Current, w/o PF; Alcohol Dependence, Lifetime (duration 312 months); Psychotic Disorder, NOS, Lifetime & Current; Low Suicide Risk, Current; |
| MDD100 | Major Depressive Disorder, Single Episode, Current, w/o PF; |
| MDD103 | Major Depressive Disorder, Single Episode, Current, w/o Psychotic Features; Low Suicide Risk, Current (not an Axis I); |
| MDDfe3 | Major Depressive Disorder, Current, Single Episode, w/o PF; Low Suicide Risk, Current (not Axis I); PTSD, Lifetime & Current; Substance Dependence, Lifetime & Current (codeine and marijuana); Adult ADHD; Bipolar NOS, Lifetime & Current; |
| MDD113 | Major Depressive Disorder, Current, Single Episode, w/o Psychotic Features; Specific Phobia, Lifetime & Current (driving); |
| MDDfe4 | Major Depressive Disorder, Single Episode, Current; Low Suicide Risk, Current (not Axis I); |
| MDD121 | Depression NOS, Past; Major Depressive Disorder, Current, Single Episode, w/o PF; Low Suicide Risk, Current; Alcohol Dependence, Lifetime & Current (duration 120 months); Substance Dependence, Lifetime & Current (heroin & cocaine); Adult ADHD; |
| MDD123 | Major Depressive Disorder, Current, Single Episode, w/o PF; Moderate Suicide Risk, Current; Social Phobia, Lifetime & Current; Alcohol Dependence, Lifetime (duration 204 months); Substance Dependence, Lifetime (speed); Substance Abuse, Lifetime (marijuana); |
| MDD124 | Major Depressive Disorder, Single Episode, Current, w/o PF; Alcohol Dependence, Lifetime & Current (132 months); |
| MDD125 | Major Depressive Disorder, Single Episode, Current, w/o PF; Social Phobia, Lifetime & Current; Low Suicide Risk, Current (not Axis I); |
| MDD126 | Major Depressive Disorder, Single Episode, Current, w/o psychotic features; Alcohol Abuse, Lifetime (duration 120 months); |
| MDD127 | Major Depressive Disorder, Single Episode, Current, w/o psychotic features; Alcohol Dependence, Lifetime & Current (duration 12 months); Cannabis Dependence, Lifetime; |
| MDD128 | Major Depressive Disorder, Current & Chronic, w/o PF; Moderate Suicide Risk, Current (not Axis I); Panic Disorder, Lifetime & Current, w/o Agoraphobia; Specific Phobia, Situational Type; Psychosis NOS, with Delusion of Persecution; |

**eReferences.**

1. Aihara M, Ida I, Yuuki N, et al. HPA axis dysfunction in unmediated major depressive disorder and its normalization by pharmacotherapy correlates with alteration of neural activity in prefrontal cortex and limbic/paralimbic regions. *Psychiatry Res.* 2007;155(3):245-256.
2. Bonte FJ, Trivedi MH, Devous MD Sr, et al. Occipital brain perfusion deficits in children with major depressive disorder. *J Nucl Med.* 2000;42(7):1059-1061.
3. Brody AL, Saxena S, Stoessei P, et al. Regional brain metabolic changes in patients with major depression treated with either paroxetine or interpersonal therapy: preliminary findings. *Arch Gen Psychiatry.* 2001;58(7):631-640.
4. Cao J, Chen X, Chen J, et al. Resting-state functional MRI of abnormal baseline brain activity in young depressed patients with and without suicidal behavior. *J Affect Disord.* 2016;205:252-263.
5. Chen G, Bian H, Jiang D, et al. Pseudo-continuous arterial spin labeling imaging of cerebral blood perfusion asymmetry in drug-naïve patients with first-episode major depression. *Biomed Rep.* 2016;5(6):675-680.
6. Chen JD, Liu F, Xun GL, et al. Early and late onset, first-episode, treatment-naïve depression: same clinical symptoms, different regional neural activities. *J Affect Disord.* 2012;143(1-3):56-63.
7. Drevets WC, Videen TO, Price JL, Preskorn SH, Carmichael ST, Raichie ME. A functional anatomical study of unipolar depression. *J Neurosci.* 1992;12(9):3628-3641.
8. Du L, Wang J, Meng B, et al. Early life stress affects limited regional brain activity in depression. *Sci Rep.* 2016;6:25338.
9. Duhameau B, Ferré JC, Jannin P, et al. Chronic and treatment-resistant depression: a study using arterial spin labeling perfusion MRI at 3Tesla. *Psychiarty Res*. 2010;182(2):111-116.
10. Fang J, Mao N, Jiang X, Li X, Wang B, Wang Q. Functional and Anatomical Brain Abnormalities and Effects of Antidepressant in Major Depressive Disorder: Combined Application of Voxel-Based Morphometry and Amplitude of Frequency Fluctuation in Resting State. *J Comput Assist Tomogr.* 2015;39(5):766-773.
11. Germain A, Nofzinger EA, Meltzer CC, et al. Diurnal variation in regional brain glucose metabolism in depression. *Biol Psychiatry.* 2007;62(5):438-445.
12. Gong Y, Hao L, Zhang X, et al. Case-control resting-state fMRI study of brain functioning among adolescents with first-episode major depressive disorder. *Shanghai Arch Psychiatry.* 2014;26(4):207-215.
13. Guo W, Liu F, Yu M, et al. Functional and anatomical brain deficits in drug-naïve major depressive disorder. *Prog Neuropsychopharmacol Biol Psychiatry.* 2014;54:1-6.
14. Guo WB, Sun XL, Liu L, et al. Disrupted regional homogeneity in treatment-resistant depression: a resting-state fMRI study. *Prog Neuropsychopharmacol Biol Psychiatry*. 2011;35(5):1297-1302.
15. Guo WB, Liu F, Chen JD, et al. Abnormal neural activity of brain regions in treatment-resistant and treatment-sensitive major depressive disorder: a resting-state fMRI study. *J Psychiatry Res*. 2012;46(10):1366-1373.
16. Guo WB, Liu F, Xue ZM, et al. Alterations of amplitude of low-frequency fluctuations in treatment-resistant and treatment-response depression: a resting-state fMRI study. *Prog Neuropsychopharmacol Biol Psychiatry.* 2012;37(10:153-160.
17. Guo WB, Liu F, Xun GL, et al. Reversal alterations of amplitude of low-frequency fluctuations in early and late onset, first-episode, drug-naïve depression. *Prog Neuropsychopharmacol Biol Psychiatry.* 2013;40:153-159.
18. Ho TC, Wu J, Shin DD, et al. Altered cerebral perfusion in executive, affective, and motor networks during adolescent depression. *J Am Acad Child Adolesc Psychiatry*. 2013;52(10):1076-1091.
19. Huang M, Lu S, Yu L, et al. Altered fractional amplitude of low frequency fluctuation associated with cognitive dysfunction in first-episode drug-naïve major depressive disorder patients. *BMC Psychiatry*. 2017;17(1):11.
20. Ito H, Kawashima R, Awata S, et al. Hypoperfusion in the limbic system and prefrontal cortex in depression: SPECT with anatomic standardization technique. *J Nucl Med*. 1996;37(3):410-414.
21. Kaichi Y, Okada G, Takamura M, et al. Changes in the regional cerebral blood flow detected by arterial spin labeling after 6-week escitalopram treatment for major depressive disorder. *J Affect Disord*. 2016;194:135-143.
22. Kegeles LS, Malone KM, Slifstein M, et al. Response of cortical metabolic deficits to serotonergic challenge in familial mood disorders. *Am J Psychiatry*. 2003;160(1):76-82.
23. Kennedy SH, Evans KR, Krüger S, et al. Changes in regional brain glucose metabolism measured with positron emission tomography after paroxetine treatment of major depression. *Am J Psychiatry*. 2001;158(6):899-905.
24. Kimbrell TA, Ketter TA, George MS, et al. Regional cerebral glucose utilization in patients with a range of severities in unipolar depression. *Biol Psychiatry*. 2002;51(3):237-252.
25. Kohn Y, Freedman N, Lester H, et al. 99mTc-HMPAO SPECT study of cerebral perfusion after treatment with medication and electroconvulsive therapy in major depression. *J Nucl Med.* 2007;48(8):1273-1278.
26. Krausz Y, Freedman N, Lester H, et al. Brain SPECT study of common ground between hypothyroidism and depression. *Int J Neuropsychopharmacol.* 2007;10(1):99-106.
27. Lai CH, Wu YT. The patterns of fractional amplitude of low-frequency fluctuations in depression patients: the dissociation between temporal regions and fronto-parietal regions. *J Affect Disord.* 2015;175:441-445.
28. Liang MJ, Zhou Q, Yang KR, et al. Identify Changes of Brain Regional Homogeneity in Bipolar Disorder and Unipolar Depression Using Resting-State fMRI. *PLoS One.* 2013;8(12):e79999.
29. Liu CH, Ma X, Wu X, et al. Resting-state brain activity in major depressive disorder patients and their siblings. *J Affect Disord.* 2013;149(1-3):299-306.
30. Liu J, Ren L, Womer FY, et al. Alterations in amplitude of low frequency fluctuation in treatment-naïve major depressive disorder measured with resting-state fMRI. *Hum Brain Mapp.* 2014;35(10):4979-4988.
31. Liu Y, Zhao X, Cheng Z, et al. Regional homogeneity associated with overgeneral autobiographical memory of first-episode treatment-naïve patients with major depressive disorder in the orbitofrontal cortex: A resting-state fMRI study. *J Affect Disord.* 2017;209:163-168.
32. Liu Z, Xu C, Xu Y, et al. Decreased regional homogeneity in insula and cerebellum: a resting-state fMRI study in patients with major depression and subjects at high risk for major depression. *Psychiatry Res.* 2010;182(3):211-215.
33. Lui S, Parkes LM, Huang X, et al. Depressive disorders: focally altered cerebral perfusion measured with arterial spin-labeling MR imaging. *Radiology.* 2009;251(2):476-484.
34. Monkul ES, Silva LAP, Narayana S, et al. Abnormal Resting State Corticolimbic Blood Flow in Depressed Unmedicated Patients With Major Depression: A 15O-H2O PET Study. *Hum Brain Mapp.* 2012;33(2):272-279.
35. Oda K, Okubo Y, Ishida R, et al. Regional cerebral blood flow in depressed patients with white matter magnetic resonance hyperintensity. *Biol Psychiatry.* 2003;52(2):150-156.
36. Perico CA, Skaf CR, Yamada A, et al. Relationship between regional cerebral blood flow and separate symptom clusters of major depression: a single photon emission computed tomography study using statistical parametric mapping. *Neurosci Lett.* 2005;384(3):265-270.
37. Qiu H, Li X, Zhao W, et al. Electroconvulsive Therapy-Induced Brain Structural and Functional Changes in Major Depressive Disorders: A Longitudinal Study. *Med Sci Monit.* 2016;22:4577-4586.
38. Shen T, Qiu M, Li C, et al. Altered spontaneous neural activity in first-episode, unmedicated patients with major depressive disorder. *Neuroreport.* 2014;25(16):1302-1307.
39. Skaf CR, Yamada A, Garrido GE, et al. Psychotic symptoms in major depressive disorder are associated with reduced regional cerebral blood flow in the subgenual anterior cingulated cortex: a voxel-based single photon emission computed tomography (SPECT) study. *J Affect Disord.* 2002;68(2-3):295-305.
40. Smith GS, Kramer E, Ma Y, et al. The functional neuroanatomy of geriatric depression. *Int J Geriatr Psychiatry.* 2009;24(8):798-808.
41. Tadayonnejad F, Yang S, Kumar A, Ajilore O. Clinical, cognitive, and functional connectivity correlations of resting-state intrinsic brain activity alterations in unmedicated depression. *J Affect Disord.* 2015;172:241-250.
42. Vardi N, Freedman N, Lester H, et al. Hyperintensities on T2-weighted imaged in the basal ganglia of patients with major depression: cerebral perfusion and clinical implications. *Psychiatry Res.* 2011;192(2):125-130.
43. Wang L, Dai W, Su Y, et al. Amplitude of Low-Frequency Oscillations in First-Episode, Treatment-Naïve Patients with Major Depressive Disorder: A Resting-State Functional MRI Study. *PLoS One.* 2012;7(10):e48658.
44. Wang L, Li K, Zhang Q, et al. Short-term effects of escitalopram on regional brain function in first-episode drug-naïve patients with major depressive disorder assessed by resting-state functional magnetic resonance imaging. *Psychol Med.* 2014;44(7):1417-1426.
45. Wang LJ, Kuang WH, Xu JJ, Lei D, Yang YC. Resting-state brain activation correlates with short-time antidepressant treatment outcome in drug-naïve patients with major depressive disorder. *J Int Med Res.* 2014;42(4):966-975.
46. Wu QZ, Li DM, Kuang WH, et al. Abnormal regional spontaneous neural activity in treatment-refractory depression revealed by resting-state fMRI. *Hum Brain Mapp.* 2011;32(8):1290-1299.
47. Xue S, Wang X, Wang W, Liu J, Qiu J. Frequency-dependent alterations in regional homogeneity in major depression. *Behav Brain Res.* 2016;306:13-19.
48. Yamaura T, Okamoto Y, Okada G, et al. Association of thalamic hyperactivity with treatment-resistant depression and poor response in early treatment for major depression: a resting-state fMRI study using fractional amplitude of low-frequency fluctuations. *Transl Psychiatry.* 2016;6:e754.
49. Yang X, Ma X, Li M, et al. Anatomical and functional brain abnormalities in unmedicated major depressive disorder. *Neuropsychiatr Dis Treat.* 2015;11:2415-2423.
50. Yang Y, Zhong N, Imamura K, et al. Task and Resting-State fMRI Reveal Altered Salience Response to Positive Stimuli in Patients with Major Depressive Disorder. *PLoS One.* 2016;11(5):e0155092.
51. Yao Z, Wang L, Lu Q, Liu H, Teng G. Regional homogeneity in depression and its relationship with separate depressive symptom clusters: a resting-state fMRI study. *J Affect Disord.* 2009;115(3):430-438.
52. Yue Y, Yuan Y, Hou Z, Jiang W, Bai F, Zhang Z. Abnormal functional connectivity of amygdala in late-onset depression was associated with cognitive deficits. *PLoS One.* 2013;8(9):e75058.
53. Zhang K, Liu Z, Cao X, et al. Amplitude of low-frequency fluctuations in first-episode, drug-naïve depressive patients: A 5-year retrospective study. *PLoS One.* 2017;12(4):e0174564.
54. Zhang X, Di X, Lei H, et al. Imbalanced spontaneous brain activity in orbitofrontal-insular circuits in individuals with cognitive vulnerability to depression. *J Affect Disord.* 2016;198:56-63.
55. Zhang X, Tang Y, Maletic-Savatic M, et al. Altered neuronal spontaneous activity correlated with glutamate concentration in medial prefrontal cortex of major depressed females: An fMRI-MRS study. *J Affect Disord.* 2016;201:153-161.
56. Alemany S, Mas A, Goldberg X, et al. Regional gray matter reductions are associated with genetic liability for anxiety and depression: an MRI twin study. *J Affect Disord.* 2013;149(1-3):175-181.
57. Arnone D, McKie S, Elliott R, et al. State-dependent changes in hippocampal grey matter in depression. *Mol Psychiatry.* 2013;18(12):1265-1272.
58. Arnone D, Pegg EJ, McKie S, et al. Decreased fronto-limbic gray matter volume associated with recurrent major depressive disorder. *European Neuropsychopharmacology.* 2009;19:s371.
59. Bergouignan L, Chupin M, Czechowska Y, et al. Can voxel based morphometry, manual segmentation and automated segmentation equally detect hippocampal volume differences in acute depression? *Neuroimage.* 2009;45(1):29-37.
60. Cai Y, Liu J, Zhang L, et al. Gray matter volume abnormalities in patients with bipolar I depressive disorder and unipolar depressive disorder: a voxel-based morphometry study. *Neurosci Bull.* 2015;31(1):4-12.
61. Chaney A, Carballedo A, Amico F, et al. Effect of childhood maltreatment on brain structure in adult patients with major depressive disorder and healthy participants. *J Psychiatry Neurosci.* 2014;39(1):50-59.
62. Cheng YQ, Xu J, Chai P, et al. Brain volume alteration and the correlations with the clinical characteristics in drug-naïve first-episode MDD patients: a voxel-based morphometry study. *Neurosci Lett.* 2010;480(1):30-34.
63. Frodl TS, Koutsouleris N, Bottlender R, et al. Depression-related variation in brain morphology over 3 years: effects of stress? *Arch Gen Psychiatry.* 2008:65(10):1156-1165.
64. Gong Q, Wu Q, Scarpazza C, et al. Prognostic predication of therapeutic response in depression using high-field MR imaging. *Neuroimage.* 2011;55(4):1497-1503.
65. Grieve SM, Korgaonkar MS, Koslow SH, Gordon E, Williams LM. Widespread reductions in gray matter volume in depression. *Neuroimage Clin.* 2013;3:332-339.
66. Hwang JP, Lee TW, Tsai SJ, et al. Cortical and subcortical abnormalities in late-onset depression with history of suicide attempts investigated with MRI and voxel-based morphometry. *J Geriatr Psychiatry Neurol.* 2010;23(3):171-184.
67. Inkster B, Rao AW, Ridler K, et al. Structural brain changes in patients with recurrent major depressive disorder presenting with anxiety symptoms. *J Neuroimaging.* 2011;21(4):375-382.
68. Kim MJ, Hamilton JP, Gotlib IH. Reduced Caudate gray Matter Volume in Women with Major Depressive Disorder. *Psychiatry Res.* 2008;164(2):114-122.
69. Lai CH, Wu YT. The gray matter alterations in major depressive disorder and panic disorder: Putative differences in the pathogenesis. *J Affect Disord.* 2015;186:1-6.
70. Lee HY, Tae WS, Yoon HK, et al. Demonstration of decreased gray matter concentration in the midbrain encompassing the dorsal raphe nucleus and the limbic subcortical regions in major depressive disorder: an optimized voxel-based morphometry study. *J Affect Disord.* 2011;133(1-2):128-136.
71. Li CT, Lin CP, Chou KH, et al. Structural and cognitive deficits in remitting and non-remitting recurrent depression: a voxel-based morphometric study. *Neuroimage.* 2010;50(1);347-356.
72. Liu CH, Jing B, Ma X, et al. Voxel-based morphometry study of the insular cortex in female patients with current and remitted depression. *Neuroscience.* 2014;262:190-199.
73. Machino A, Kunisato Y, Matsumoto T, et al. Possible involvement of rumination in gray matter abnormalities in persistent symptoms of major depression: an exploratory magnetic resonance imaging voxel-based morphometry study. *J Affect Disord.* 2014;168:229-35.
74. Mak AK, Wong MM, Han SH, Lee TM. Gray matter reduction associated with emotion regulation in female outpatients with major depressive disorder: a voxel-based morphometry study. *Prog Neuropsychopharmacol Biol Psychiatry.* 2009;33(7):1184-1190.
75. Peng J, Liu J, Nie B, et al. Cerebral and cerebellar gray matter reduction in first-episode patients with major depressive disorder: a voxel-based morphometry study. *Eur J Radiol.* 2011;80(2):395-399.
76. Perico CAM, Duran FL, Zanetti MV, et al. A population-based morphometric MRI study in patients with first-episode psychotic bipolar disorder: comparison with geographically matched healthy controls and major depressive disorder subjects. *Bipolar Disord.* 2011;13(1):28-40.
77. Redlich R, Almeida JJ, Grotegerd D, et al. Brain morphometric biomarkers distinguishing unipolar and bipolar depression. A voxel-based morphometry-pattern classification approach. *JAMA Psychiatry.* 2014;71(11):1222-1230.
78. Salvadore G, Nugent AC, Lemaitre H, et al. Prefrontal cortical abnormalities in currently depressed versus currently remitted patients with major depressive disorder. *Neuroimage.* 2011;54(4):2643-2651.
79. Scheuerecker J, Meisenzahl EM, Koutsouleris N, et al. Orbitofrontal volume reductions during emotion recognition in patients with major depression. *J Psychiatry Neurosci.* 2010;35(5);311-320.
80. Serra-Blasco M, Portella MJ, Gomez-Anson B, et al. Effects of illness duration and treatment resistance on gray matter abnormalities in major depression. *Br J Psychiatry.* 2013;202:434-440.
81. Shad MU, Muddasani S, Rao U. Gray Matter Differences Between Healthy and Depressed Adolescents: A Voxel-Based Morphometry Study. *J Child Adolesc Psychopharmacol.* 2012;22(3);190-197.
82. Shah PJ, Ebmeier KP, Glabus MF, Goodwin GM. Cortical grey matter reductions associated with treatment-resistant chronic unipolar depression. Controlled magnetic resonance imaging study. *Br J Psychiatry.* 1998;172:527-532.
83. Soriano-Mas C, Hernandez-Ribas R, Pujol J, et al. Cross-sectional and longitudinal assessment of structural brain alterations in melancholic depression. *Biol Psychiatry.* 2011;69(4):318-325.
84. Stratmann M, Konrad C, Kugel H, et al. Insular and hippocampal gray matter volume reductions in patients with major depressive disorder. *PLoS One.* 2014;9(7):e102692.
85. Tang Y, Wang F, Xie G, et al. Reduced ventral anterior cingulate and amygdala volumes in medication-naïve females with major depressive disorder: A voxel-based morphometric magnetic resonance imaging study. *Psychiatry Research: Neuroimaging.* 2007;156(1):83-86.
86. van Eijndhoven P, van Wingen G, Katzenbauer M, et al. Paralimbic cortical thickness in first-episode depression: evidence for trait-related differences in mood regulation. *Am J Psychiatry.* 2013;170(12):1477-1486.
87. van Tol MJ, van der Wee NJ, van den Heuvel OA, et al. Regional brain volume in depression and anxiety disorders. *Arch Gen Psychiatry.* 2010;67(20):1002-1011.
88. van Tol MJ, Li M, Metzger CD, et al. Local cortical thinning links to resting-state disconnectivity in major depressive disorder. *Psychol Med.* 2014;44(10):2053-2065.
89. Vasic N, Walter H, Höse A, Wolf RC. Gray matter reduction associated with psychopathology and cognitive dysfunction in unipolar depression: a voxel-based morphometry study. *J Affect Disord.* 2008;109(1-2):107-116.
90. Wagner G, Koch K, Schachtzabel C, Schultz CC, Sauer H, Schlösser RG. Structrual brain alterations in patients with major depressive disorder and high risk for suicide: evidence for a distinct neurobiological entity? *Neuroimage.* 2011;54(2):1607-1614.
91. Zhang X, Yao S, Zhu X, Wang x, Zhu X, Zhong M. Gray matter volume abnormalities in individuals with cognitive vulnerability to depression: A voxel-based morphometry study. *J Affect Disord.* 2012;136(3):443-452.
92. Zou K, Deng W, Li T, et al. Changes of brain morphometry in first-episode, drug-naïve, non-late-life adult patients with major depression: an optimized voxel-based morphometry study. *Biol Psychiatry.* 2010;67(2):186-188.
